# The Leader Peptide peTrpL Forms Antibiotic-Containing Ribonucleoprotein Complexes for Posttranscriptional Regulation of Multiresistance Genes

**DOI:** 10.1128/mBio.01027-20

**Published:** 2020-06-16

**Authors:** Hendrik Melior, Sandra Maaß, Siqi Li, Konrad U. Förstner, Saina Azarderakhsh, Adithi R. Varadarajan, Maximilian Stötzel, Muhammad Elhossary, Susanne Barth-Weber, Christian H. Ahrens, Dörte Becher, Elena Evguenieva-Hackenberg

**Affiliations:** a Institute of Microbiology and Molecular Biology, University of Giessen, Giessen, Germany; b Institute of Microbiology, University of Greifswald, Greifswald, Germany; c ZB MED—Information Centre for Life Sciences, University of Cologne, Cologne, Germany; d Agroscope & SIB Swiss Institute of Bioinformatics, Wädenswil, Switzerland; Université de Sherbrooke; University of Würzburg

**Keywords:** antimicrobial compound, leader peptide, multidrug resistance, nucleoprotein complex, posttranscriptional regulation, *Agrobacterium tumefaciens*, *Alphaproteobacteria*, *Bradyrhizobium*, *Sinorhizobium meliloti*, antibiotic resistance, posttranscriptional RNA-binding protein, posttranscriptional control mechanisms, ribonucleoprotein complex, transcription attenuator

## Abstract

Bacterial ribosome-dependent attenuators are widespread posttranscriptional regulators. They harbor small upstream open reading frames (uORFs) encoding leader peptides, for which no functions in *trans* are known yet. In the plant symbiont Sinorhizobium meliloti, the tryptophan biosynthesis gene *trpE(G)* is preceded by the uORF *trpL* and is regulated by transcription attenuation according to tryptophan availability. However, *trpLE(G)* transcription is initiated independently of the tryptophan level in S. meliloti, thereby ensuring a largely tryptophan-independent production of the leader peptide peTrpL. Here, we provide evidence for a tryptophan-independent role of peTrpL in *trans*. We found that peTrpL increases the resistance toward tetracycline, erythromycin, chloramphenicol, and the flavonoid genistein, which are substrates of the major multidrug efflux pump SmeAB. Coimmunoprecipitation with a FLAG-peTrpL suggested *smeR* mRNA, which encodes the transcription repressor of *smeABR*, as a peptide target. Indeed, upon antibiotic exposure, *smeR* mRNA was destabilized and *smeA* stabilized in a peTrpL-dependent manner, showing that peTrpL acts in the differential regulation of *smeABR*. Furthermore, *smeR* mRNA was coimmunoprecipitated with peTrpL in antibiotic-dependent ribonucleoprotein (ARNP) complexes, which, in addition, contained an antibiotic-induced antisense RNA complementary to *smeR*. *In vitro* ARNP reconstitution revealed that the above-mentioned antibiotics and genistein directly support complex formation. A specific region of the antisense RNA was identified as a seed region for ARNP assembly *in vitro*. Altogether, our data show that peTrpL is involved in a mechanism for direct utilization of antimicrobial compounds in posttranscriptional regulation of multiresistance genes. Importantly, this role of peTrpL in resistance is conserved in other *Alphaproteobacteria*.

## Abstract (precis)

Leader peptides encoded by transcription attenuators are widespread small proteins that are considered nonfunctional in *trans*. We found that the leader peptide peTrpL of the soil-dwelling plant symbiont Sinorhizobium meliloti is required for differential, posttranscriptional regulation of a multidrug resistance operon upon antibiotic exposure. Multiresistance achieved by efflux of different antimicrobial compounds ensures survival and competitiveness in nature and is important from both evolutionary and medical points of view. We show that the leader peptide forms antibiotic- and flavonoid-dependent ribonucleoprotein complexes (ARNPs) for destabilization of *smeR* mRNA encoding the transcription repressor of the major multidrug resistance operon. The seed region for ARNP assembly was localized in an antisense RNA, whose transcription is induced by antimicrobial compounds. The discovery of ARNP complexes as new players in multiresistance regulation opens new perspectives in understanding bacterial physiology and evolution and potentially provides new targets for antibacterial control.

## INTRODUCTION

Multidrug-resistant bacteria pose an increasing problem; therefore, the discovery of new resistance mechanisms is of great interest (1, 2). Multidrug resistance is mediated by efflux pumps capable of extruding different antibacterial drugs (antibiotics) (1, 3). Known multiresistance mechanisms are exemplified by the Escherichia coli membrane transporter AcrB and the Pseudomonas putida transcription repressor TtgR. The multidrug resistance (MDR) inner membrane transporter AcrB harbors distal and proximal binding pockets, which can accommodate unrelated antibiotics (1, 4–6). The TetR-type repressor TtgR can bind different antibiotics using two overlapping binding sites, the first broader and hydrophobic and the second deeper and with polar residues. Upon ligand binding, TtgR changes its confirmation and falls off the promoter of an MDR efflux pump operon (7–9).

Soil bacteria are a prominent reservoir of resistance mechanisms, since many antibiotic producers are also living in soil (10, 11). Particularly, plant-interacting bacteria have powerful efflux pumps, which can also extrude plant antimicrobials (8, 11–13). The MDR efflux pump SmeAB is the major efflux pump of our model organism, the soil-dwelling plant symbiont Sinorhizobium meliloti (12). It was shown that deletion of the *smeR* repressor gene, which is located immediately downstream of *smeAB*, increases the nodulation competitiveness of S. meliloti and its multidrug resistance (12). The *smeAB* and *smeR* genes are probably cotranscribed, since in a related alphaproteobacterium, the plant pathogen Agrobacterium tumefaciens, the homologous genes are located in the *acrABR* operon (13). In contrast, in E. coli, the *acrR* repressor gene is not cotranscribed with the MDR pump encoding genes *acrAB* (1). Cotranscription of the repressor and structural genes poses a challenge for their regulation. Upon antibiotic exposure, it is expected that repressor synthesis should be avoided in order to ensure increased efflux pump production. Differential regulation of cotranscribed genes can be achieved posttranscriptionally, at the level of RNA (14, 15).

Known RNA-based mechanisms for regulation of antibiotic resistance or susceptibility include noncoding RNAs and RNA-binding proteins. In *cis*, antibiotic-induced translation inhibition at short upstream open reading frames (uORFs) relieves transcription or translation attenuation of downstream resistance genes in Gram-positive bacteria (16–18). Furthermore, *cis*-acting antisense RNAs (asRNAs), *trans*-acting small RNAs (sRNAs), and the RNA chaperone Hfq directly or indirectly regulate bacterial resistance (18–25). Additionally, small proteins may also be important for resistance.

Small proteins (≤50 amino acids [aa]), despite carrying out important functions, are poorly characterized or not included in genome annotations (26, 27). Examples of important small proteins are ribosomal protein L34 (46 and 44 aa in E. coli and Bacillus subtilis, respectively [28, 29]), the Bacillus subtilis 26-aa protein SpoVM needed for endospore formation (30), and the E. coli 49-aa protein AcrZ, which interacts with AcrB and selectively enhances the AcrAB-TolC pump export (31). The aforementioned uORFs in attenuators (17, 32–34) are common sources of small proteins, the bacterial leader peptides (usually <20 aa). However, no examples for leader peptides acting in *trans* are known yet.

**FIG 1:**
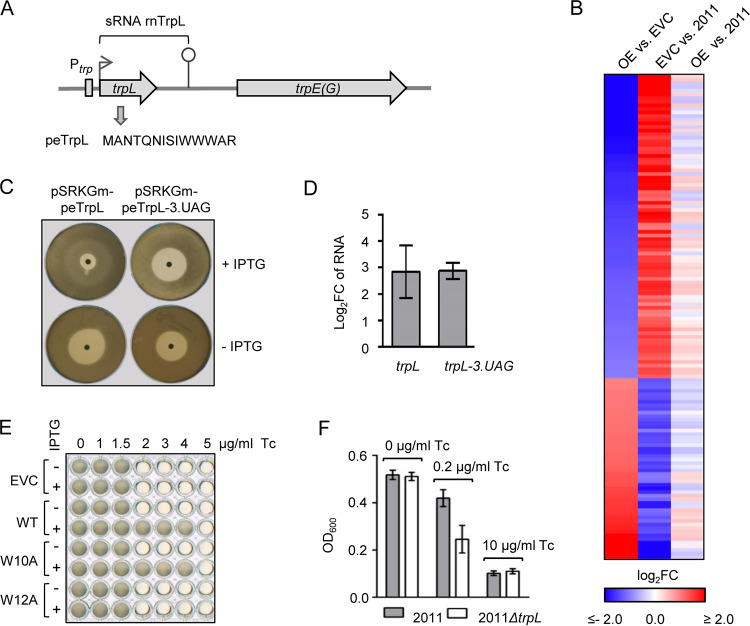
The leader peptide peTrpL increases the resistance to Tc. (A) Scheme of the S. meliloti *trpLE(G)* locus. The transcription start site and transcription terminator of the *trp* attenuator are depicted by a flexed arrow and a hairpin, respectively. The *trans*-acting products of the *trp* attenuator, the sRNA rnTrpL, and the leader peptide peTrpL, are indicated. (B) Heat map with RNA-seq data of the following strains: OE, overexpressing strain 2011 (pRK-rnTrpL); EVC, empty vector control 2011 (pRK4352); 2011, parental strain. The heat map shows results for 135 genes with strong differences (log_2_ fold change [FC] >2.0 or <−2.0) in the comparisons of OE versus EVC and EVC versus 2011, which had no or low differences (log_2_ FC >−0.5 or <0.5) in the comparison of OE versus 2011 (see Data Set S1 in the supplemental material). (C) Representative agar plates with zones of growth inhibition by centrally applied Tc. S. meliloti 2011 Δ*trpL* harboring the indicated plasmids was used. Presence of IPTG in the agar is indicated. (D) qRT-PCR analysis of the increase in *trpL* and *trpL-3.UAG* mRNA levels 10 min after IPTG addition to liquid cultures of strains used in panel C, in comparison to the EVC. (E) Growth of S. meliloti 2011 Δ*trpL* harboring pSRKGm (EVC) or its derivatives allowing for production of peTrpL (WT), peTrpL-W10A, or peTrpL-W12A in microtiter plates, in medium with increasing Tc concentrations (given at the top). Presence of IPTG in the medium is indicated. Shown is a representative plate with final bacterial growth. (F) OD_600_ reached overnight by the indicated strains in microtiter plates, in medium containing the given Tc concentrations. The graphs show data from three independent cultures (mean ± standard deviation).

A widespread class of ribosome-dependent transcription attenuators regulates amino acid biosynthesis genes in Gram-negative bacteria. The best-studied example is the attenuator of the tryptophan (Trp) biosynthesis operon, which contains the small uORF *trpL* harboring several consecutive Trp codons (32, 34). S. meliloti has three *trp* operons, of which only *trpE(G)* is regulated by transcription attenuation (Fig. 1A) (35). Upon *trpL* translation in the nascent RNA, the attenuator can adopt two mutually exclusive structures. Under conditions of Trp shortage, ribosomes transiently stall at the Trp codons, leading to the formation of an antiterminator structure and the structural genes are expressed. Conversely, when enough Trp is available, *trpL* translation at the Trp codons is fast, the transcription terminator is formed, and expression of the structural genes is abolished (32, 34, 35).

10.1128/mBio.01027-20.6 DATA SET S1 RNA-seq analysis of S. meliloti strain 2011 (WT), the empty vector control (EVC) 2011 (pRK4352), and the constitutively overexpressing (OE) strain 2011 (pRK-rnTrpL). Download Data Set S1, XLSX file, 0.4 MB. Copyright © 2020 Melior et al. 2020 Melior et al. This content is distributed under the terms of the Creative Commons Attribution 4.0 International license.

Recently it was shown that the S. meliloti sRNA rnTrpL, which is generated by *trpE(G)* transcription attenuation, acts in *trans* to destabilize *trpDC* mRNA (36). Since the 5′ end of rnTrpL starts with the ATG codon of the *trpL* small ORF (sORF) encoding the 14-aa leader peptide peTrpL (Fig. 1A) (35), it may act as a small leaderless mRNA in addition to its role as a riboregulator. In contrast to E. coli, where transcription of the *trp* genes is repressed under high Trp conditions (34), in S. meliloti, the *trpLE(G)* operon is not subjected to transcription repression (36). Thus, *trpLE(G)* is constitutively transcribed during growth, suggesting that peTrpL is produced independently of Trp availability and may have adopted Trp-independent function(s).

Here, we show that in S. meliloti, the leader peptide peTrpL (14 aa) has a role in multidrug resistance. We found that peTrpL is involved in the antibiotic-dependent destabilization of *smeR* mRNA, which encodes the TtgR-type repressor of the major MDR efflux pump SmeAB. Moreover, we show that peTrpL forms antibiotic-dependent complexes with *smeR* mRNA and an asRNA, which is induced upon antibiotic exposure. Thus, we uncovered unexpected interactions of antimicrobial compounds with the leader peptide peTrpL and target RNAs.

(This article and its previous version were submitted to an online preprint archive [37, 38].)

## RESULTS

### The leader peptide peTrpL increases the resistance to tetracycline.

The starting point of this study was our observation that ectopic constitutive overproduction of the attenuator sRNA rnTrpL (which harbors the ORF *trpL*) from plasmid pRK-rnTrpL apparently counteracts transcriptome-wide effects of tetracycline (Tc) in S. meliloti. This observation was based on transcriptome sequencing (RNA-seq) analysis of the overexpressing strain 2011 (pRK-rnTrpL), the empty vector control (EVC) strain 2011 (pRK4352), and the parental strain 2011 (strain 2011 was grown in tryptone-yeast extract [TY] medium with streptomycin [Sm], the strains harboring Tc-resistance plasmids [pRK series] in the presence of Sm and Tc). A comparison of the overexpressing strain with the EVC revealed significant changes in the levels of thousands of RNAs (Fig. 1B; see also Data Set S1 in the supplemental material). Surprisingly, when the EVC was compared to strain 2011, inverse changes were observed. Consistently, the transcriptomes of the overexpressing strain and the parental strain 2011 were quite similar (Fig. 1B).

A possible explanation for the differences between the EVC and strain 2011 is a general effect of Tc on mRNA translation (39). If so, Fig. 1B suggests that the Tc effect in the overexpressing strain is much lower than in the EVC. Therefore, we hypothesized that overproduction of the sRNA rnTrpL and/or peTrpL peptide encoded by this sRNA may lead to a lower Tc concentration in the overexpressing cells and thus to a higher resistance to Tc. To address this, we used the deletion mutant strain 2011 Δ*trpL*, which lacks the native rnTrpL RNA being transcribed from the chromosome (36). To test whether the peTrpL peptide is responsible for the increased resistance, we constructed plasmid pSRKGm-peTrpL, which allows for isopropyl-β-d-thiogalactopyranoside (IPTG)-inducible peTrpL production. As a negative control, a plasmid was constructed in which the third codon of the ORF was replaced with a stop codon (pSRKGm-peTrpL-3.UAG). On plates with centrally applied Tc, the zone of growth inhibition of strain 2011 Δ*trpL* (pSRKGm-peTrpL) was much smaller when IPTG was added to the agar medium. In contrast, the diameter of the bacterium-free halo of the negative control was not decreased on IPTG-containing plates (Fig. 1C). Lower *trpL-3.UAG* mRNA levels were excluded as one possible reason for the failure to increase resistance. (Fig. 1D). The results suggested that peTrpL is necessary and sufficient for increased resistance to Tc. To provide further support for the role of peTrpL, peptides with specific amino acid residue substitutions compared to wild-type (WT) peTrpL were used. Growth experiments in liquid cultures revealed that the mutated peptide peTrpL-W10A still increased the Tc resistance, while peTrpL-W12A was nonfunctional (Fig. 1E). Furthermore, peTrpL also increased the resistance of S. meliloti to two natural tetracyclines, chlortetracycline and oxytetracycline (see Fig. S1A and B).

10.1128/mBio.01027-20.1 FIG S1 Characterization of peTrpL. (A) Representative plates with zones of growth inhibition of caused by the centrally applied chlortetracycline or oxytetracycline. S. meliloti 2011 Δ*trpL* containing the indicated plasmids was used. Presence of IPTG in the growth medium is indicated. (B) Data from panel A and two additional independent cultures. (C) qRT-PCR analysis of changes in the *smeR* mRNA levels 10 min after IPTG addition to cultures of S. meliloti 2011 harboring pRK4352 and one of the following plasmids: pSRKGm (EVC), pSRKGm-peTrpL (WT), pSRKGm-peTrpL-W10A (W10A), or pSRKGm-peTrpL-W12A (W12A). (D) Fluorescence of S. meliloti 2011 containing *egfp* reporter fusions. P_rrn_-*trpL-egfp*, a leaderless *trpL-egfp* mRNA (containing the first 6 *trpL* codons fused to the third *egfp* codon) was constitutively transcribed from P*_rrn_* of plasmid pRK-trpL-egfp (36); P_rrn_-SD-*trpL-egfp*, typical Shine-Dalgarno (SD) sequence was present between the P_rrn_ promoter and the ATG of *trpL*; P_sinI_-*trpL-egfp* and P_sinI_-SD-*trpL-egfp*, pSW2-based, Gm-resistance plasmids harboring the constitutive P_sinI_ promoter instead of P_rrn_ were used; P_sinI_-SD-*egfp*, control construct for *egfp* expression lacking the *trpL* codons, the complete *egfp* gene was preceded by the SD sequence. Strains harboring pRK plasmids were incubated overnight without Tc, before 20 μg/ml Tc was added. To strains with pSW2 plasmids, 1.5 μg/ml Tc was added. Fluorescence was measured 10 min after Tc addition and before the addition. The results show a peTrpL′-EGFP accumulation upon Tc exposure, which was independent of the used heterologous promoter and the leaderless status of the mRNA. This could be explained by the assumption that the six peTrpL amino acids of the peTrpL′-EGFP fusion protein stabilize it in the presence of Tc. (E and F) Northern blot hybridization for mRNA half-live analysis. The half-lives of *smeR*, *smeA*, and *smeB* were analyzed in 2011 Δ*trpL* (pSRKGm-peTrpL) cultures. Addition of IPTG is indicated; Tc was not added. The data complement the results shown in Fig. 3F and G. Therefore, to both cultures, instead of Tc, the solvent ethanol was added. Ten minutes after IPTG and ethanol addition, rifampin was added to stop cellular transcription. At the indicated time points after rifampin addition (top), culture aliquots were withdrawn and RNA was isolated. Detected mRNAs (specificity of used probes) are indicated on the left. 16S rRNA was used as a loading control. The calculated half-lives are given at the bottom. All graphs show data from three independent experiments, presented as means ± standard deviations. Download FIG S1, PDF file, 0.4 MB. Copyright © 2020 Melior et al. 2020 Melior et al. This content is distributed under the terms of the Creative Commons Attribution 4.0 International license.

Next, we compared the growth of strains 2011 and 2011 *ΔtrpL* at different Tc concentrations. The strains grew similarly in the absence of Tc and failed to grow in medium containing 10 μg/ml Tc, i.e., one-half the concentration used in our selective medium. However, in medium supplemented with 0.2 μg/ml Tc, the parental strain 2011 reached a significantly higher optical density at 600 nm (OD_600_) than the 2011 Δ*trpL* mutant (Fig. 1F), providing evidence that *trpL* is important for the intrinsic resistance of S. meliloti to Tc.

We also analyzed the peTrpL levels in rich TY medium with and without Tc by mass spectrometry. The peptide was detected in cultures of strain 2011 during growth without Tc and, in line with its role in resistance, accumulated at a factor of 91.9 ± 18.5 at 10 min after addition of 1.5 μg/ml Tc (for detailed mass spectrometry [MS] results, see the PRIDE repository with the data set identifier PXD018342). Constitutively transcribed *trpL*::*egfp* fusions revealed that this accumulation is regulated posttranscriptionally (Fig. S1D). As it is known that in E. coli leaderless mRNAs are preferentially translated under stress (40), we constructed additional *trpL*::*egfp* fusions harboring a Shine-Dalgarno (SD) sequence. However, the peTrpL accumulation was the same for leaderless constructs or constructs with an SD (Fig. S1D). Thus, peTrpL accumulation in response to Tc might be regulated at the protein level.

### peTrpL is involved in the posttranscriptional regulation of the *smeABR* operon.

**FIG 2:**
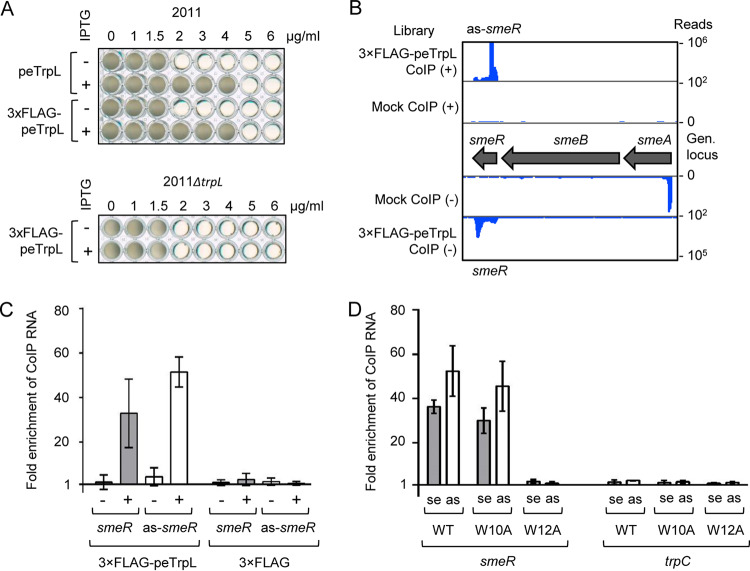
CoIP with 3×FLAG-peTrpL identifies *smeR* mRNA and its asRNA as Tc-dependent peTrpL targets. (A) Growth of strains 2011 (pSRKGm-peTrpL) and 2011 (pSRKGm-3×FLAG-peTrpL) (top), and 2011 Δ*trpL* (pSRKGm-3×FLAG-peTrpL) (bottom) in microtiter plates. IPTG presence and peptide products are indicated on the left. For other descriptions, see the legend for Fig. 1E. (B) Integrated Genome Browser view of the *smeABR* locus with mapped cDNA reads of the RNA-seq analysis of RNA, which was coimmunoprecipitated from strain 2011 (pSRKGm-3×FLAG-peTrpL, pRK4352) 10 min after peptide induction. Mock CoIP, strain 2011 (pSRKGm-peTrpL, pRK4352) was used. Tc was present in the growth medium (20 μg/ml) and in the CoIP washing buffer (2 μg/ml). Shown is representative data from one of three independent experiments. (C) qRT-PCR analysis showing the enrichment of *smeR* mRNA and as-*smeR* RNA in CoIPs with 3×FLAG-peTrpL or 3×FLAG peptide. Presence of Tc (2 μg/ml) in the washing buffer is indicated. Two-plasmid strains containing pRK4352 and one of the plasmids pSRKGm-3×FLAG-peTrpL or pSRKGm-3×FLAG was used. (D) qRT-PCR analysis showing the enrichment of the indicated RNAs in CoIPs with S. meliloti 2011 producing 3×FLAG-peTrpL (WT), 3×FLAG-peTrpL-W10A (W10A), or 3×FLAG-peTrpL-W12A (W12A) in the absence of plasmid pRK4352; 1.5 μg/ml Tc was present in the washing buffer. se, sense RNA; as, asRNA. The graphs show data from three independent cultures (mean ± standard deviation). RNA enrichment was calculated in comparison to the mock CoIP.

To address the mechanism by which peTrpL influences resistance, we aimed to coimmunoprecipitate it along with its interaction partner(s) using an N-terminally tagged 3×FLAG-peTrpL. Induced 3×FLAG-peTrpL production increased the Tc resistance in the parental but not in Δ*trpL* background (Fig. 2A), suggesting that the tagged peptide is functional but only acts in conjunction with the native peptide. Therefore, coimmunoprecipitation (CoIP) with FLAG-directed antibodies was conducted in the parental background.

We hypothesized that the peTrpL peptide may need Tc for interaction with other macromolecules in the cell. Therefore, first, a two-plasmid strain containing the gentamicin (Gm) resistance plasmid for peptide production, and the empty Tc resistance plasmid pRK4352 was cultivated in medium with 20 μg/ml Tc and used for CoIP with antibodies coupled to magnetic beads. The samples were divided in two: one half was washed with a buffer containing 2 μg/ml Tc (corresponds to the MIC), and the other half was washed with a buffer without Tc. Coimmunoprecipitated RNA and proteins were analyzed. For a control CoIP, a 3×FLAG peptide was used. Furthermore, a mock CoIP was performed with a strain in which peTrpL was induced instead of 3×FLAG-peTrpL or 3×FLAG peptide.

In contrast to the control CoIPs, in the 3×FLAG-peTrpL CoIP, much more RNA was coimmunoprecipitated when the beads were washed with Tc in the buffer (routinely 1.8 to 2.2 μg) than without Tc (routinely 40 to 80 ng). RNA-seq revealed that RNA corresponding to three genomic loci was strongly enriched with 3×FLAG-peTrpL (37), one of them being the *smeR* mRNA that encodes the repressor of the *smeAB* genes (Fig. 2B). In contrast, many different proteins were coimmunoprecipitated with 3×FLAG-peTrpL when Tc was absent from the washing buffer (see Fig. S2). Mass spectrometry analyses suggested that in the presence of Tc, peTrpL might interact with unknown proteins, while in the absence of Tc, many cellular proteins bound nonspecifically to peTrpL (see Data Set S2), probably due to complex disassembly and loss of peptide structure (the pure peptide is disordered; see SP-2 in reference 41). Furthermore, the mass spectrometry data revealed that in the presence of Tc, native peTrpL was coimmunoprecipitated with the FLAG-tagged peTrpL, pointing to peptide dimerization or oligomerization (see Data Set S3).

10.1128/mBio.01027-20.2 FIG S2 Tricine SDS-PAGE analysis of coimmunoprecipitated proteins. (A) Silver-stained gel showing coimmunoprecipitated proteins. After washing the antibody-coupled beads with a buffer containing Tc (Tc+) or with buffer devoid of Tc (Tc−), SDS loading buffer was added and SDS-PAGE was performed. 3×FLAG-peTrpL CoIP, strain 2011 (pSRKGm-3×FLAG-peTrpL, pRK4352) was used. Mock CoIP, strain 2011 (pSRKGm-peTrpL, pRK4352) was used. Strains were grown in medium supplemented with Gm and Tc. The CoIP was conducted 10 min after induction of 3×FLAG-peTrpL or peTrpL production from the respective plasmid with IPTG. While essentially no proteins were coimmunoprecipitated in the mock control, many more proteins were coimmunoprecipitated with the 3×FLAG-peTrpL peptide when the beads were washed with buffer without Tc. In the Tc+ sample, the major band presumably corresponding to 3×FLAG-peTrpL was more intense than in the Tc− sample (marked with an arrow). (B) Silver-stained gel showing results from a control CoIP performed with strain 2011 (pSRKGm-3×FLAG, pRK4352), in which production of a 3×FLAG peptide was induced with IPTG. See also the description for panel A. No difference was observed between samples from beads washed in the presence or absence of Tc. (C and D) Coomassie-stained gel used for mass spectrometry analysis. Analyzed bands are marked with arrows in panel D. The mass spectrometry results are summarized in Data Set S2. See also the description for panel A. (E) Western blot analysis of CoIP samples. FLAG-directed antibodies were used. See also the description for panel A. The result suggests that most of the 3×FLAG-peTrpL peptide was lost during washing the beads in the absence of Tc (compare to Data Set S3), and/or 3×FLAG-peTrpL was instable in the Tc− sample. The molecular weight of marker proteins (lane M) is given in kDa. Download FIG S2, PDF file, 0.3 MB. Copyright © 2020 Melior et al. 2020 Melior et al. This content is distributed under the terms of the Creative Commons Attribution 4.0 International license.

10.1128/mBio.01027-20.7 DATA SET S2 Mass spectrometry analysis of SDS-PAGE bands shown in Fig. S2D. Download Data Set S2, XLSX file, 0.1 MB. Copyright © 2020 Melior et al. 2020 Melior et al. This content is distributed under the terms of the Creative Commons Attribution 4.0 International license.

10.1128/mBio.01027-20.8 DATA SET S3 Mass spectrometry analysis of peptide amounts in CoIP samples and in MS2-MBP affinity chromatography elution fractions. Download Data Set S3, XLSX file, 0.1 MB. Copyright © 2020 Melior et al. 2020 Melior et al. This content is distributed under the terms of the Creative Commons Attribution 4.0 International license.

Since the CoIP identified *smeR* mRNA as a possible peTrpL interaction partner related to multidrug resistance, it was analyzed in more detail. Phyre^2^ analysis (42) revealed that SmeR is similar to TtgR (99.9% confidence; 93% coverage), the P. putida repressor capable of binding different antibiotics (8). Surprisingly, we observed that not only *smeR* mRNA, but also the corresponding asRNA (which we named as-*smeR* RNA) was coimmunoprecipitated with 3×FLAG-peTrpL (Fig. 2B). Both RNAs were coimmunoprecipitated only in the presence of Tc, and importantly, they were not coimmunoprecipitated with the 3×FLAG control (Fig. 2C). Furthermore, although residual *smeA* RNA was detected in the mock control, it was not enriched by the CoIP with 3×FLAG-peTrpL (Fig. 2B). We conclude that the peTrpL part of 3×FLAG-peTrpL was responsible for the Tc-dependent CoIP of *smeR* and as-*smeR* RNA.

For the next experiments, plasmid pRK4352 was omitted and Tc was added to the cultures at a subinhibitory concentration (1.5 μg/ml) prior to CoIP. In addition to 3×FLAG-peTrpL, FLAG-tagged peptides with W10A or W12A replacements were also used. Both the *smeR* mRNA and as-*smeR* RNA coimmunoprecipitated with 3×FLAG-peTrpL and 3×FLAG-peTrpL-W10A but not with 3×FLAG-peTrpL-W12A (Fig. 2D). This result confirmed the importance of the W12 residue for peptide function and that CoIP of *smeR* mRNA and as-*smeR* RNA does not depend on pRK4352. Furthermore, a control mRNA (*trpC*) was not enriched, demonstrating the specificity of the CoIP.

**FIG 3:**
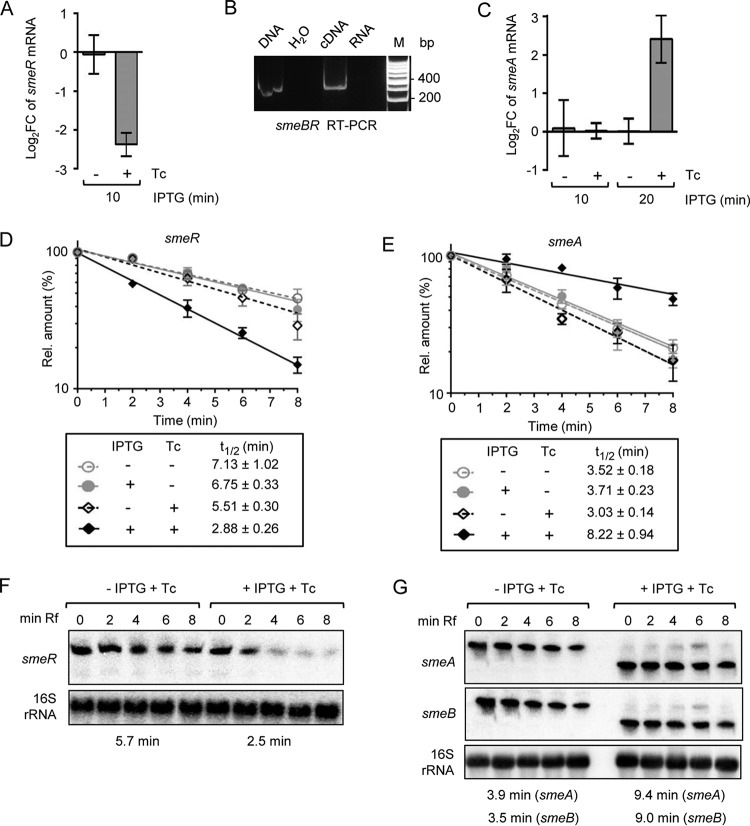
peTrpL and Tc are involved in the differential posttranscriptional regulation of *smeABR.* (A) qRT-PCR analysis of changes in the *smeR* levels 10 min after IPTG addition to two parallel 2011 Δ*trpL* (pSRKGm-peTrpL) cultures. Tc (1.5 μg/ml) was added together with IPTG to one of the cultures (indicated). (B) RT-PCR analysis with a forward primer located in *smeB* and reverse primer located in *smeR*. The PCR template input is indicated at the top. (C) Changes in the *smeA* levels 10 and 20 min after IPTG addition. See also descriptions for panel A. (D and E) mRNA stability determination by qRT-PCR using *smeR* and *smeA* specific primer pairs. To 2011 Δ*trpL* (pSRKGm-peTrpL) cultures, IPTG and/or Tc (1.5 μg/ml) was added and 10 min thereafter, rifampin was added. The relative mRNA level values after stop of transcription by rifampin were determined and plotted against the time. The calculated half-lives are indicated. (F and G) Northern blot analysis of RNA from the experiments described for panels D and E. RNAs detected by the used probes are indicated on the left side. 16S rRNA was used as a loading control. The conditions used and time after rifampin addition are given at the top, the calculated half-lives at the bottom (see also Fig. S1 in the supplemental material). Ten minutes after IPTG and Tc addition (0 min in respect to rifampin addition), the tricistronic *smeABR* mRNA was detected with the *smeR*-specific probe (internally radiolabeled 128-nt *in vitro* transcript) but not with the *smeA*- and *smeB*-directed probes (radiolabeled DNA probes generated by random priming). This could be explained by the higher sensitivity and stronger binding of the RNA probe. In all graphs, data from three independent cultures are presented as means ± standard deviations.

To test the influence of peTrpL and Tc on *smeR*, we analyzed by quantitative reverse transcriptase PCR (qRT-PCR) changes in the *smeR* mRNA levels 10 min postinduction (p.i.) of the peptide in two parallel 2011 Δ*trpL* (pSRKGm-peTrpL) cultures. To one of them, 1.5 μg/ml Tc was added together with IPTG. The *smeR* mRNA level was decreased only if Tc was applied (Fig. 3A). In line with the above-described data, the W12 residue was critical for *smeR* downregulation by the peptide (Fig. S1C). The *smeR* gene is located downstream of *smeAB* (see Fig. 2B) and is cotranscribed with *smeB* (Fig. 3B), suggesting a tricistronic *smeABR* mRNA as described for the homologous *acrABR* in A. tumefaciens (13). Therefore, we also analyzed the *smeA* mRNA level and found an increase 20 min (but not 10 min) p.i. of peTrpL, provided Tc was also added to the cultures (Fig. 3C).

The observed changes in the mRNA levels could be explained by changed mRNA stability. Indeed, using qRT-PCR, we detected a decreased *smeR* and increased *smeA* stability 10 min p.i. of peTrpL, but only in the presence of Tc (Fig. 3D and E). Northern blot hybridization confirmed peTrpL- and Tc-dependent *smeR* destabilization in the *smeABR* cotranscript and suggested that this destabilization converts *smeABR* to a shorter and more stable *smeAB* transcript (Fig. 3F and G; see also Fig. S1). In summary, these results show that both peTrpL and Tc are involved in the differential posttranscriptional regulation of the *smeABR* operon.

### peTrpL increases multiresistance and forms antibiotic-dependent ribonucleoprotein complexes.

**FIG 4:**
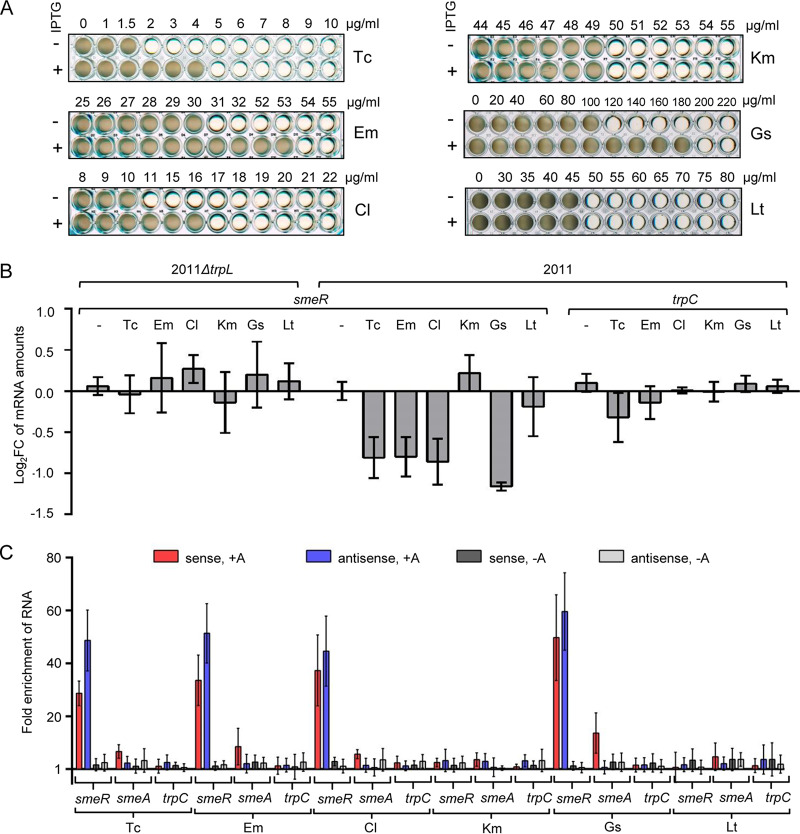
peTrpL increases multidrug resistance and forms antibiotic-dependent ribonucleoprotein (ARNP) complexes. (A) Growth of strain 2011 Δ*trpL* (pSRKGm-peTrpL) in microtiter plates. The increasing concentrations of the antibiotics and flavonoids are given at the top (μg/ml). The antimicrobial compounds are indicated on the right and IPTG presence on the left. Shown are representative plates with final growth. (B) qRT-PCR analysis of changes in the *smeR* levels 10 min after addition of the indicated antibiotics and flavonoids (used at subinhibitory concentrations) to cultures of strains 2011 Δ*trpL* and 2011. *trpC*, control mRNA. (C) qRT-PCR analysis of enrichment of the indicated RNAs by CoIP with 3×FLAG-peTrpL in comparison to the mock CoIP. Antibiotics and flavonoids (used at subinhibitory concentrations), which were added together with IPTG to cultures of S. meliloti 2011 containing either pSRKGm-3×FLAG-peTrpL or pSRKGm-peTrpL (mock CoIP), are indicated at the bottom. Presence (+A) or absence (−A) of the antibiotics or flavonoids in the washing buffer are indicated at the top. RNA enrichment was calculated in comparison to the mock CoIP. In all graphs, data from three independent cultures are presented as means ± standard deviations.

According to reference 12, the antibiotics Tc, erythromycin (Em), chloramphenicol (Cl), and the flavonoid genistein (Gs) are substrates of the MDR efflux pump SmeAB, while kanamycin (Km) and the flavonoid luteolin (Lt) are not. We tested whether peTrpL affects the resistance of S. meliloti against these antimicrobial compounds. Indeed, induced peTrpL production increased the resistance to the SmeAB substrates, but not to Km and Lt (Fig. 4A). Moreover, peTrpL induction increased the cellular efflux (see Fig. S3).

10.1128/mBio.01027-20.3 FIG S3 Induced peTrpL production increases the cellular efflux. (A) Nile red efflux assay for determination of efflux activity. Strains 2011 (pSRKGm-peTrpL, pRK4352) and the EVC 2011 (pSRKGm, pRK4352) were grown in the presence or absence of IPTG. The Nile red dye generates a fluorescence signal only if present in the cell. First CCCP was added to the cultures to stop cellular efflux, allowing subsequently added Nile Red to accumulate in the cells. After removing CCCP to reverse the inhibition of cellular efflux, glucose was added as energy source to start the efflux immediately before measuring fluorescence intensity. Fast decline of fluorescence over time correlates with high efflux pump activity. Data from a representative experiment are shown. (B) Tc competes with Nile red for the efflux, showing that the same pump(s) extrudes them. Nile red efflux assay was performed with strain 2011 (pSRKGm-peTrpL, pRK4352) cultivated with IPTG. Increasing Tc concentrations were added together with glucose, which was used to start the efflux. According to our data, the peTrpL-dependent increase of cellular efflux can be contributed to the SmeAB MDR efflux pump. Whether the TetA pump encoded by pRK4352 is influenced by peTrpL remains to be analyzed in the future. Download FIG S3, PDF file, 0.3 MB. Copyright © 2020 Melior et al. 2020 Melior et al. This content is distributed under the terms of the Creative Commons Attribution 4.0 International license.

These results are in line with a function of peTrpL in regulation of multiresistance and suggest that the above-described SmeAB substrates may participate in the peTrpL-dependent *smeR* downregulation. To test this, we exposed strains 2011 and 2011 Δ*trpL* to subinhibitory concentrations of the antimicrobial compounds for 10 min and analyzed *smeR* mRNA by qRT-PCR. Indeed, exposure of strain 2011 to Tc, Em, Cl, and Gs led to an *smeR* decrease, while Km and Lt had no effect (Fig. 4B). The control mRNA *trpC* was essentially not affected, showing the specificity of *smeR* downregulation upon exposure to SmeAB substrates. Importantly, the *smeR* decrease was not observed in strain 2011 Δ*trpL*, confirming the involvement of peTrpL in this regulation (Fig. 4B).

Next, we used strain 2011 (pSRKGm-3×FLAG-peTrpL) to test whether the SmeAB substrates support CoIP of 3×FLAG-peTrpL with *smeR* mRNA and its asRNA. Indeed, upon exposure to Tc, Em, Cl, or Gs, and provided the respective antimicrobial compound was present in the washing buffer, *smeR* mRNA and its asRNA were strongly enriched by the CoIP (Fig. 4C). The CoIP specificity is clearly shown by the failure to enrich the control mRNA *trpC*. In comparison to that for *smeR*, the *smeA* mRNA was enriched only very weakly in the presence of the SmeAB substrates, probably because of *smeABR* cotranscription. Km and Lt did not lead to CoIP of the analyzed RNAs (Fig. 4C). These results suggest the existence of antibiotic-dependent ribonucleoprotein (ARNP) complexes comprising peTrpL, *smeR* mRNA, as-*smeR* RNA, and one of the antibiotics Tc, Em, Cl, or the flavonoid Gs.

### *In vitro* analysis of ARNP complexes reveals a key role of the asRNA.

**FIG 5:**
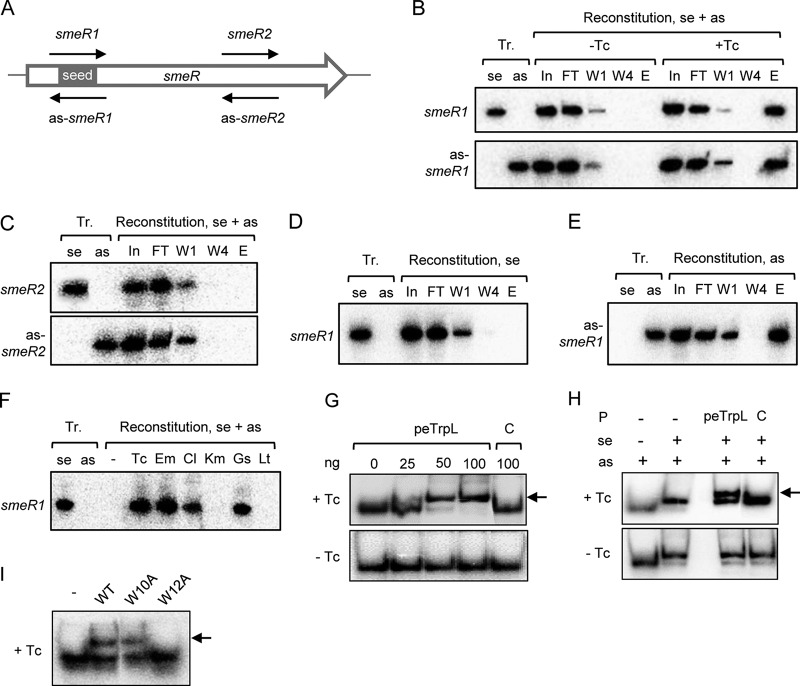
Reconstitution of ARNP complexes. (A) Scheme of the *smeR* ORF (white arrow) showing the proposed peTrpL binding seed region and the *in vitro* transcripts used for ARNP reconstitution (thin black arrows). (B to F) Northern blot analyses with probes detecting the *in vitro* transcripts indicated on the left side of each panel. At the top of each panel, the loaded samples are indicated. Tr., *in vitro* transcripts were loaded as hybridization and size controls; se, sense transcript (corresponds to *smeR* mRNA); as, antisense transcript (corresponds to as-*smeR* RNA); reconstitution se + as, both sense and antisense transcript were present in the reconstitution reaction; reconstitution se, only the sense transcript was used for reconstitution; reconstitution as, only the antisense transcript was used for reconstitution. The 50-μl reconstitution samples were used for CoIP and following fractions were loaded on the gel (volume or amount loaded): In, input fraction (5 μl); FT, flowthrough (5 μl); W1 and W4, first and last washing fractions (10 μl each); E, elution fraction, 1/10 of the purified CoIP-RNA. Shown are representative results. (B) ARNP reconstitution using the sense transcript *smeR1* and the antisense transcript as-*smeR1*, which correspond to the putative peTrpL binding site (seed region). Addition of Tc is indicated. Top, hybridization with a probe directed against *smeR1.* Bottom, rehybridization of the membrane with a probe directed against as-*smeR1*. (C), Reconstitution with the control transcripts *smeR2* and as-*smeR2* in the presence of Tc. Top, hybridization with a probe directed against *smeR2.* Bottom, rehybridization of the membrane with a probe directed against as-*smeR2*. (D) ARNP reconstitution using only *smeR1* in the presence of Tc. (E) ARNP reconstitution using only as-*smeR1* in the presence of Tc. (F) ARNP reconstitution using *smeR1*, as-*smeR1*, and the indicated antibiotics and flavonoids. Only elution fractions were loaded. −, representative negative control of reconstitution, 2 μl ethanol was added to the reconstitution mixture. (G to I) EMSAs in the presence or absence of Tc (indicated on the left) using radioactively labeled as-*smeR1* and 10% PAA gels. Shift caused by peptide is indicated by an arrow. (G) shift of as-*smeR1* by increasing peTrpL amounts (indicated in nanograms). C, control, unrelated protein was used. (H) Shift of an RNA duplex (*smeR1* and as-*smeR1* transcripts) by 50 ng peTrpL. Presence of transcripts and proteins in the loaded samples is indicated at the top. (I) WT peTrpL or peptides (50 ng) with the indicated amino acid exchanges were used in the EMSAs with as-*smeR1*. −, no protein was present.

To study ARNP complex assembly *in vitro*, we performed reconstitution using synthetic components. Figure 2B (see above) shows a high 70-nucleotide (nt) peak in the RNA-seq data of the coimmunoprecipitated as-*smeR* RNA. We reasoned that this peak may correspond to the binding site of peTrpL and synthesized a corresponding *in vitro* transcript named as-*smeR1*. A complementary *smeR1* transcript was also synthesized, which corresponds to a part of *smeR* mRNA (Fig. 5A). The two transcripts were mixed with synthetic WT peTrpL and 3×FLAG-peTrpL. Samples with and without Tc were prepared. After incubation, reconstituted complexes were coimmunoprecipitated and analyzed by Northern blotting hybridization. Both *smeR1* and as-*smeR1* transcripts were coimmunoprecipitated only from the Tc-containing samples (see the elution fractions in Fig. 5B), indicating successful reconstitution of an antibiotic-dependent complex. In a control experiment with Tc, transcripts *smeR2* and as-*smeR2* corresponding to a downstream part of *smeR* (Fig. 5A) were used. These control transcripts were not coimmunoprecipitated (and were thus not detected in the elution fraction) (Fig. 5C). Thus, the seed for ARNP assembly is contained in as-*smeR1* and/or *smeR1*.

Next, we tested whether single-stranded RNA is sufficient for ARNP formation. When used without an asRNA in reconstitution reactions, the *smeR1* transcript was not coimmunoprecipitated with 3×FLAG-peTrpL (Fig. 5D). In contrast, when as-*smeR1* was used alone for reconstitution, it was coimmunoprecipitated (Fig. 5E), showing that as-*smeR1* contains the direct binding site of peTrpL and/or Tc in the ARNP. We also tested whether other antimicrobial compounds support ARNP reconstitution using *smeR1* and as-*smeR1* transcripts. The *smeR1* transcript was coimmunoprecipitated only if one of the SmeAB substrates was added (Fig. 5F).

To further validate the Tc-dependent interaction of as-*smeR1* with peTrpL and to show that 3×FLAG-peTrpL is not needed for this interaction, an electrophoretic mobility shift assay (EMSA) was conducted. The radioactively labeled transcript was shifted by increasing peTrpL concentrations only in the presence of Tc (Fig. 5G). In addition, we performed EMSA using both sense and asRNA and detected a Tc-dependent shift of the RNA duplex by peTrpL (Fig. 5H). Synthetic mutated peptides were also used, showing the importance of the W12 residue for the interaction with the asRNA (Fig. 5I).

The key role of the asRNA prompted us to test whether MS2-tagged asRNA can be used for ARNP purification (43). MS2-as-*smeR* RNA was induced from a plasmid in the 2011 background, together with 3×FLAG-peTrpL. After MS2-MBP affinity chromatography in the presence or absence of Tc, the elution fractions were analyzed by mass spectrometry. Both 3×FLAG-peTrpL and native peTrpL were detected only in fractions obtained in the presence of Tc (Data Set S3). These results confirm (i) a role of the asRNA in the ARNP complex, (ii) the Tc-dependence of the complex, and (iii) peptide dimerization or oligomerization.

### The as-*smeR* RNA is induced by substrates of the SmeAB efflux pump.

**FIG 6:**
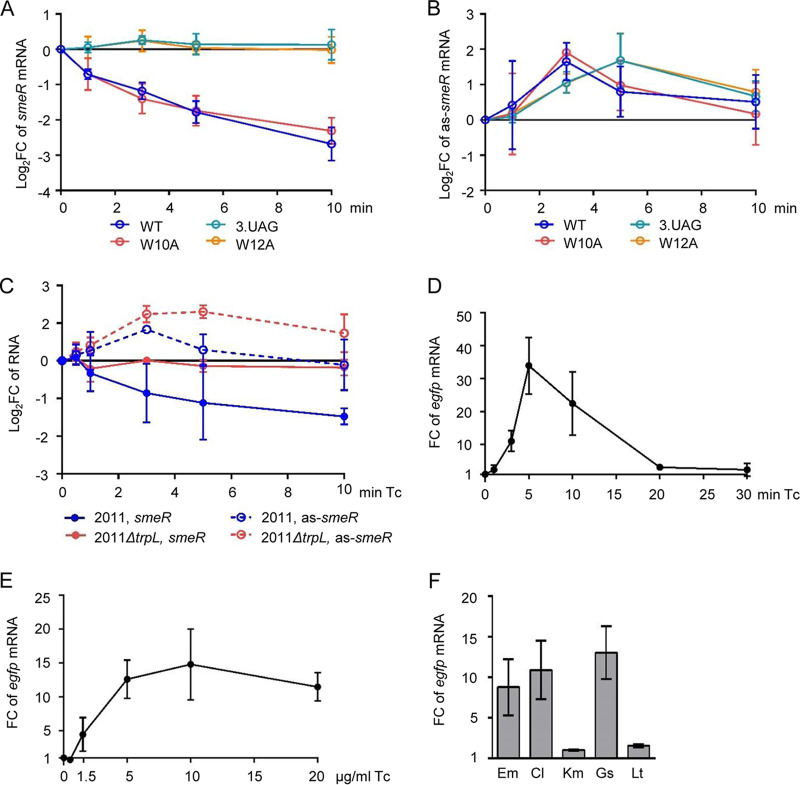
The as-*smeR* RNA is induced by substrates of the SmeAB efflux pump. (A) Kinetics of changes in the level of *smeR* mRNA at 1, 3, 5, and 10 min after addition of IPTG and 1.5 μg/ml Tc to cultures of S. meliloti 2011 Δ*trpL* harboring one of the following plasmids: pSRKGm-peTrpL (WT), pSRK-Gm-3.UAG (3.UAG), pSRKGm-peTrpL-W10A (W10A), or pSRKGm-peTrpL-W12A (W12A), as determined by qRT-PCR. Changes were calculated in comparison to the EVC. (B) Kinetics of changes in the level of as-*smeR* RNA. See also the description for panel A. (C) Kinetics of changes in the level of *smeR* mRNA and as-*smeR* RNA at 1, 3, 5, and 10 min after addition of 1.5 μg/ml Tc to cultures of strains 2011 and 2011 Δ*trpL*, as determined by qRT-PCR. Changes were calculated in comparison to the cultures to which the solvent ethanol was added instead of Tc. (D to F) qRT-PCR analysis of reporter *egfp* mRNA reflecting P_as_ promoter activity. (D) Changes in the *egfp* level upon addition of 20 μg/ml Tc to 2011 (pSUP-PasRegfp) cultures for the indicated time (min Tc). The cultures harboring the chromosomally integrated plasmid, which confers resistance to Tc, were incubated overnight in medium without Tc. No plasmid loss was detected by qPCR. (E) Changes in the *egfp* level 3 min after addition of Tc to 2011 (pSUP-PasRegfp) cultures. Used Tc concentrations are indicated. (F) Changes in the *egfp* level 3 min after addition of the indicated antibiotics and flavonoids at subinhibitory concentrations to 2011 (pSUP-PasRegfp) cultures. In all graphs, data from three independent cultures are presented as means ± standard deviations.

Despite the key role of the as-*smeR* RNA in ARNP assembly, this asRNA was not detected in a previous high-throughput study (44) nor in the RNA-seq analysis performed at the beginning of this study. We tested by qRT-PCR whether WT peTrpL, peTrpL-3.UAG (dipeptide), peTrpL-W10A, or peTrpL-W12A influence the asRNA level in the Δ*trpL* background. RNA was isolated at the time points 0, 1, 3, 5, and 10 min p.i. by IPTG and Tc addition. As expected, the *smeR* mRNA level was continuously decreased only in strains producing the functional peptides peTrpL and peTrpL-W10A (Fig. 6A). In contrast, the level of the as-*smeR* RNA was transiently increased in all strains, although the kinetics slightly differed between strains producing functional and nonfunctional peptides. (Fig. 6B). This suggested that the asRNA is induced independently of peTrpL in response to Tc.

Next, we analyzed the kinetics of *smeR* and as-*smeR* changes in strains 2011 and 2011 Δ*trpL* after Tc addition. In strain 2011, we detected a continuous decrease in the *smeR* mRNA level and a slight but statistically significant increase in the as-*smeR* RNA level 3 min (but not 10 min) after Tc addition (Fig. 6C). Importantly, in strain 2011 Δ*trpL*, in which as expected the *smeR* mRNA level was not changed, the asRNA increase was detectable even at the time point of 10 min (Fig. 6C). The results support a Tc-dependent and peTrpL-independent asRNA induction and suggest that in strain 2011, the asRNA is degraded faster and/or its induction is relieved faster (due to faster Tc efflux) than in the Δ*trpL* mutant. They also confirm the importance of *trpL* for *smeR* regulation upon Tc exposure.

To test whether an antibiotic-inducible antisense promoter (P_as_) is present downstream of *smeR*, a plasmid harboring a fusion of the putative P_as_ (from **−**290 to + 2) to *egfp*, was integrated into the chromosome of strain 2011. Upon exposure to Tc, the level of the reporter *egfp* mRNA was transiently increased, with a significant increase already at 3 min, peak at 5 min, and almost no increase at 20 min of exposure time (Fig. 6D). Probably at the last time point, Tc was already pumped out from the cells by newly synthesized plasmid-borne TetA, the chromosomally encoded SmeAB, and possibly also by other MDR efflux pumps of S. meliloti (12).

In the next experiment, P_as_ induction upon 3-min exposure to different Tc concentrations was studied (Fig. 6E), including the subinhibitory concentration of 1.5 μg/ml, which was used in many of the experiments. Additionally, 3-min exposure to different antimicrobial compounds was applied. Figure 6F shows that transcription from P_as_ was induced by Em, Cl, and Gs (but not by Km or Lt). These results strongly suggest the existence of a promoter driving the antibiotic- and flavonoid-induced transcription of as-*smeR* RNA.

### Conservation of the peTrpL role in resistance.

**FIG 7:**
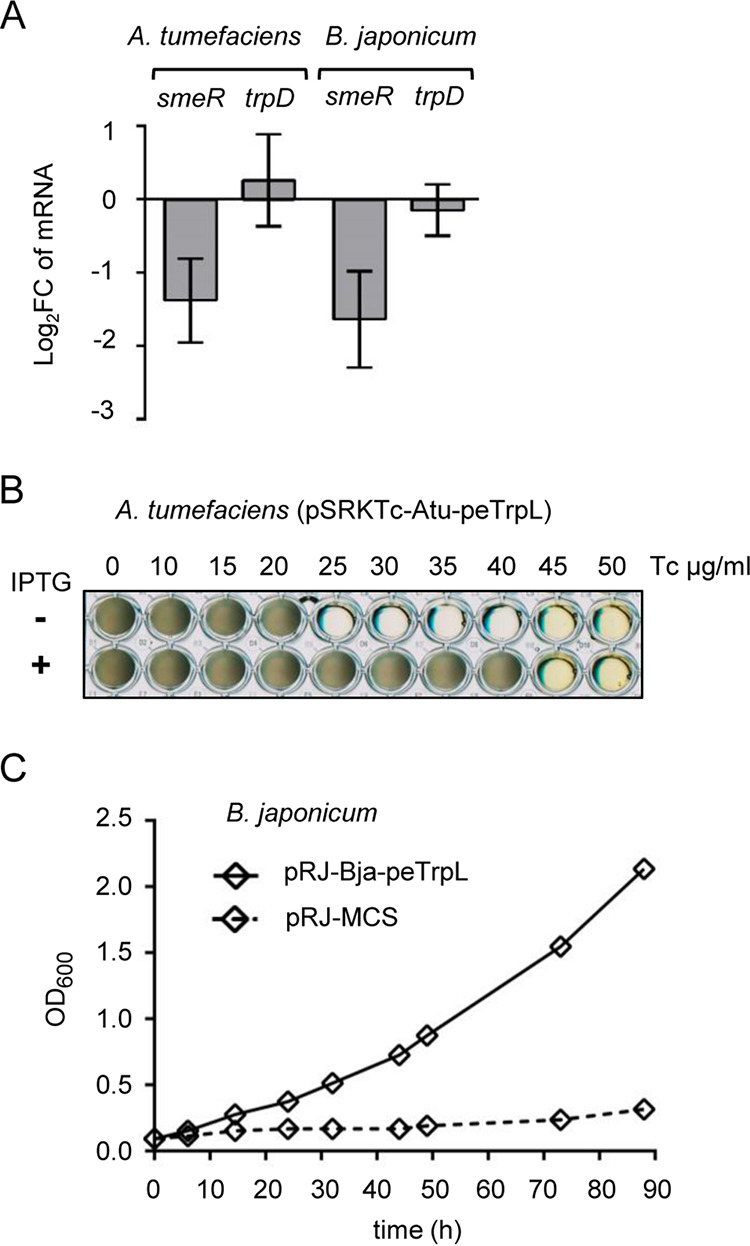
Conservation of peTrpL function in resistance. (A) qRT-PCR analysis of the expression of *smeR* homologs and *trpD* upon overproduction of the respective peTrpL homologs in A. tumefaciens and B. japonicum. Data from three independent cultures are presented as means ± standard deviations. (B) Growth of the indicated A. tumefaciens strain in microtiter plates. Presence of IPTG in the medium and the Tc concentrations used are indicated. A representative plate is shown. (C) Growth curves of B. japonicum containing the indicated plasmids (pRJ-MCS, empty vector). Medium supplemented with 100 μg/ml Tc was used. Data from three independent cultures are presented as mean ± standard deviations (smaller than the symbols in the graph).

To test whether the role of peTrpL in resistance is conserved in other bacteria, we used Agrobacterium tumefaciens (which, together with S. meliloti, belongs to the *Rhizobiaceae*), and the more distantly related Bradyrhizobium japonicum (a *Bradyrhizobiaceae* member). Besides the consecutive Trp (W) residues in their C-terminal halves, the leader peptides Atu-peTrpL (MNIVSKNIANWWWSSFLRP, 19 aa) and Bja-peTrpL (MSTAVAPARLWWRTS, 15 aa) do not show sequence conservation compared to peTrpL of S. meliloti (MANTQNISIWWWAR). Despite this, in both species, the mRNA levels of their *smeR* homologs were specifically decreased upon overproduction of the corresponding peTrpL homolog (Fig. 7A). Furthermore, the homologous overproduction of the leader peptides increased the Tc resistance of both A. tumefaciens and B. japonicum (Fig. 7B and C). Of note, production of Atu-peTrpL and Bja-peTrpL in the heterologous host S. meliloti did not increase its multidrug resistance (see Fig. S4). These results show that despite their low sequence conservation, the alphaproteobacterial peTrpL peptides have a conserved role in resistance.

10.1128/mBio.01027-20.4 FIG S4 Heterologous peTrpL peptides do not increase the multiresistance of S. meliloti. Shown are representative plates with final growth of strain 2011 Δ*trpL* containing the indicated plasmids. The antimicrobial compounds used, their concentrations, and presence of IPTG in the medium are indicated. Download FIG S4, PDF file, 0.4 MB. Copyright © 2020 Melior et al. 2020 Melior et al. This content is distributed under the terms of the Creative Commons Attribution 4.0 International license.

## DISCUSSION

In this study, we show a proof of principle for a bacterial leader peptide exerting a function in *trans*. We provide strong evidence for the role of peTrpL, which is the leader peptide of the Trp biosynthesis gene *trpE(G)*, in multidrug resistance. The surprising role of peTrpL in a Trp-unrelated mechanism could be explained by the lack of Trp-dependent transcription repression of *trpLE(G)* in S. meliloti (35, 36). Similarly to the *leu* operon in Salmonella enterica serovar Typhimurium, and in contrast to that of the *trp* operon in E. coli and *Salmonella*, *trpE(G)* expression in S. meliloti during growth is exclusively controlled by transcription attenuation (32, 34, 36, 45). When Trp is available, transcription between *trpL* and *trpE(G)* is terminated (34–36), but further peTrpL production is probably ensured by the sRNA rnTrpL, which harbors the *trpL* ORF. The presence of peTrpL in S. meliloti grown in rich medium and its strong accumulation upon exposure to Tc are consistent with the idea that uncoupling of *trpL* expression from Trp availability enabled peTrpL to adopt a Trp-independent function in *trans*.

**FIG 8:**
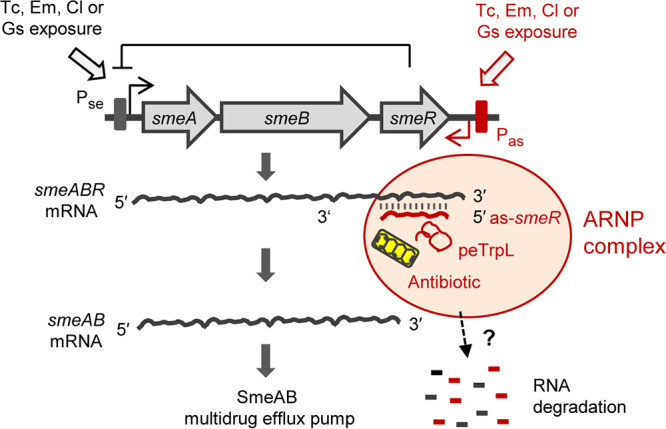
Model for the differential posttranscriptional regulation of *smeABR* by peTrpL, antimicrobial compounds, and the antisense RNA as-s*meR*. Gene *smeR* encodes the repressor of the *smeABR* operon. Upon exposure to the SmeABR substrates Tc, Em, Cl, or Gs, transcription of the tricistronic *smeABR* mRNA and as-*smeR* RNA from promoters P_se_ (12) and P_as_ (this work), respectively, is induced. The peTrpL peptide, together with one of the mentioned antibiotics or the flavonoid, forms an ARNP complex with the as-*smeR* and *smeR* RNAs, which are then degraded. This leads to *smeR* downregulation and ensures upregulation of *smeAB* encoding the major MDR efflux pump of S. meliloti.

The peTrpL-dependent increased resistance of S. meliloti to substrates of the SmeAB MDR efflux pump fits well with the identification of *smeR* as a peTrpL target. The *in vitro* reconstitution data suggest that *smeR* mRNA is an indirect target, the direct target being the as-*smeR* RNA, which is induced upon exposure to the analyzed SmeAB substrates. This induction was difficult to detect as an increase in the as-*smeR* RNA level but was easily detected using a transcription reporter mRNA. The increase in the asRNA level was transient and occurred in parallel to a continuous *smeR* mRNA decrease (Fig. 6A to C), suggesting codegradation of both RNAs (Fig. 8). Such codegradation may essentially prevent the detection of as-*smeR* despite its active transcription. The longer detection window of increased as-*smeR* levels in the Δ*trpL* background (Fig. 6D) could be attributed to lack of codegradation with *smeR* but also to less efficient Tc efflux due to lack of the peTrpL-dependent differential *smeABR* regulation.

As mentioned in the introduction, the need for uncoupling of *smeAB* and *smeR* expression upon antibiotic exposure is obvious. The here-described differential posttranscriptional *smeABR* regulation serves to downregulate SmeR synthesis by concomitant SmeAB production. Our data support the following model of peTrpL-dependent *smeABR* regulation. Upon exposure to SmeAB substrates, transcription of *smeABR* and as-*smeR* is induced. The as-*smeR* RNA forms a duplex with the *smeR* mRNA part of the tricistronic *smeABR* transcript. Additionally, a specific seed region of as-*smeR* (Fig. 5) is bound by peTrpL in an antibiotic- or flavonoid-dependent manner, leading to ARNP assembly, RNA degradation, and, as a consequence, *smeR* downregulation (Fig. 8). This model provides an example of how MDR operons, in which the repressor gene is cotranscribed with the structural genes, might be regulated at the level of RNA.

The unexpected direct involvement of antimicrobial compounds in this posttranscriptional peTrpL-mechanism is useful for bacterial adaptation, because it enables their rapid sensing at the level of RNA. Upon exposure, an antibiotic or flavonoid enters the cell and causes ARNP assembly, resulting in *smeR* downregulation and efficient production of the MDR efflux pump SmeAB. In the course of adaptation, when the intracellular antibiotic concentration is low because of efficient efflux (and/or because exposure stops), the *smeR* destabilization by peTrpL is relieved due to ARNP disassembly, the repressor SmeR is synthesized, and *smeABR* transcription is repressed again.

Based on our data, we suggest that in addition to the MDR efflux pumps and multidrug-binding TtgR-type repressors, bacteria developed a third multiresistance-related mechanism for the binding of structurally different organic molecules, which is based on ARNP complexes. We found that the Tc-dependent ARNP complex contains peptide dimers or oligomers and that the W12 residue of the 14-aa leader peptide is necessary for ARNP formation. Further details of the interaction of the leader peptide with antimicrobial compounds and target RNA remain to be uncovered. We observed conservation of peTrpL function in A. tumefaciens and B. japonicum despite low sequence conservation and inability of cross-regulation in a heterologous host (Fig. 7 and see Fig. S4 in the supplemental material). This suggests molecular adaptation of peTrpL to complex formation with antibiotics and RNA target sequences in each of the species. Interestingly, dysregulation of *trp* genes and Tc sensitivity were reported for a B. subtilis polynucleotide phosphorylase mutant, suggesting a connection between the *trp* operon and resistance in other bacteria (46).

The peTrpL-mediated resistance of S. meliloti and related bacteria is probably crucial for survival in soil, rhizosphere, and plants, where exposure to antimicrobial compounds is common. Bacterial strategies that ensure survival at high antibiotic concentrations and increase the competitiveness at subinhibitory concentrations are relevant from both evolutionary and medical points of view (47–49). Although S. meliloti is a soil bacterium with no medical relevance, it is a major model organism for studying interactions between bacteria and higher organisms (50). The mechanisms of interaction between S. meliloti and its plant hosts are similar to the mechanisms employed by the animal pathogen *Brucella* and the plant pathogen A. tumefaciens (51, 52). The identification of an attenuator leader peptide as a conserved player in the intrinsic bacterial resistance to antibiotics and the unexpected detection of ARNP complexes are interesting for two reasons: first, this new knowledge opens new perspectives in understanding bacterial physiology and evolution, and second, it potentially provides new targets for antibacterial control.

## MATERIALS AND METHODS

### Cultivation of bacteria and exposure to antimicrobial compounds.

Strains used in this work are listed in Table S1 in the supplemental material. *Sinorhizobium* (*Ensifer*) *meliloti* 2011 (53, 54), Agrobacterium tumefaciens (A. fabrum) NTL4 (pZLR4) (55, 56), and their derivatives were cultivated in TY medium (57) at 30°C; Bradyrhizobium japonicum (B. diazoefficiens) 110spc4 (58, 59) were cultured in peptone-salts-yeast extract (PSY) medium (60) at 30°C, and E. coli in was cultured in LB medium at 37°C (61). Liquid cultures of *Alphaproteobacteria* were cultivated semiaerobically (30 ml medium in a 50-ml Erlenmeyer flask at 140 rpm) to an OD_600_ of 0.5 and then processed further. For growth experiments in 96-well microtiter plates, 300 μl culture (diluted to an OD_600_ of 0.1) per well was used. Plates were incubated on the shaker (140 rpm) at 30°C for 60 h (until the cultures entered the stationary phase). At least three independent experiments were performed. IPTG was used at a final concentration of 1 mM.

10.1128/mBio.01027-20.5 TABLE S1 Strains and plasmids used in this work. Download Table S1, PDF file, 0.5 MB. Copyright © 2020 Melior et al. 2020 Melior et al. This content is distributed under the terms of the Creative Commons Attribution 4.0 International license.

The following selective antibiotic concentrations were used when resistance was encoded on a plasmid or the chromosome: tetracycline (Tc) (20 μg/ml for S. meliloti and A. tumefaciens; B. japonicum was cultivated with 25 μg/ml Tc in liquid and 50 μg/ml Tc on plates), gentamicin (Gm) (10 μg/ml in liquid cultures and 20 μg/ml in plates), streptomycin (Sm) (250 μg/ml), and spectinomycin (Sp) (100 μg/ml).

The following subinhibitory concentrations of antibiotics and flavonoids were used: 1.5 μg/ml Tc, 27 μg/ml Em, 9 μg/ml Cl, 45 μg/ml Km, 90 μg/ml Gs, and 45 μg/ml Lt. Other concentrations used are given in the figures and their legends. The time of exposure to antibiotics and flavonoids is indicated in the legends. Tc (tetracycline hydrochloride) and Km (kanamycin sulfate) were purchased from Roth (Karlsruhe, Germany). The other antibiotics, including chlortetracycline hydrochloride and oxytetracycline hydrochloride, and the flavonoids were purchased from Sigma-Aldrich.

When exposure to antibiotics was studied, two parallel cultures were used. To one of them, the respective antibiotic was added. To the second control culture (nonexposed culture), the same volume of solvent of the respective antibiotic was added. Typically, 60 μl Tc solution or ethanol was added to 30 ml culture.

For the zone of growth inhibition tests, strains 2011 Δ*trpL* (pSRKGm-peTrpL) and 2011 Δ*trpL* (pSRKGm-peTrpL-3.UAG) were used. Fifteen milliliters bottom TY agar was overlaid with 10 ml TY top agar mixed with 1 ml S. meliloti culture (OD_600_ of 0.5). The bottom and the top agar were supplemented with 20 μg/ml Gm. After solidification of the top agar, a Whatman paper disk was placed in the middle of the plate, and 5 μl Tc solution (10 μg/μl in 70% ethanol) was applied to the disk. Plates were incubated overnight at 30°C before measuring the diameter of the zone of growth inhibition. Three independent experiments were conducted.

### Cloning and conjugation.

Cloning in E. coli was performed by standard procedures (61). FastDigest restriction enzymes and Phusion polymerase (Thermo Fisher Scientific) were used. PCR amplicons were first cloned in pJet1.2/blunt (CloneJet PCR Cloning kit; Thermo Fisher Scientific) and then subcloned into conjugative plasmid. For cloning of the *trpL* ORFs with codons exchanged for synonymous codons or with mutated codons, complementary oligonucleotides were annealed and cloned directly into the desired conjugative plasmids. In comparison to the chromosomal *trpL* of S. meliloti, the recombinant sORF for production of wild-type peTrpL contained 11 nucleotide exchanges. Instead of the WT sequence ATG GCA AAC ACG CAG AAC ATT TCG ATC TGG GCT CGC TGA, the recombinant sequence ATG GCG AAC ACC CAG AAC ATC AGC ATT TGG GCC CGG TAG was used in order to avoid effects based on possible RNA-mediated regulation by base pairing with mRNA targets (36). The used synonymous codons were nonrare codons to avoid tRNA shortage in the (over)expressing strain (62). Insert-containing plasmids were analyzed by Sanger sequencing (sequencing service by Microsynth Seqlab, Göttingen, Germany) with plasmid-specific primers. The used oligonucleotides (primers) were synthesized by Microsynth (Balgach, Switzerland). They are listed in Data Set S4. The plasmids used and their characteristics are given in Table S1.

10.1128/mBio.01027-20.9 DATA SET S4 Oligonucleotides used in this work. Download Data Set S4, XLSX file, 0.1 MB. Copyright © 2020 Melior et al. 2020 Melior et al. This content is distributed under the terms of the Creative Commons Attribution 4.0 International license.

The conjugative plasmids pRK4352 (used for constitutive expression [63]), and pSRKGm and pSRKTc (both used for IPTG-inducible expression [64]) can replicate autonomously in S. meliloti and A. tumefaciens. Where indicated, two-plasmid S. meliloti strains harboring the Tc resistance-conferring empty vector pRK4352 in addition to a Gm resistance-conferring pSRKGm construct were used. The two-plasmid strains were used to guarantee bacterial growth in Tc-containing medium when peptides were produced from pSRKGm plasmids. Plasmid pSUP202pol4 (60) was used for construction of an integration vector for S. meliloti. Because of the lack of a suitable inducible plasmid for B. japonicum, for peptide overproduction in this organism, the constitutive promoter-containing integration vector pRJPaph-MCS was used (65).

To clone MS2-as-smeR under the control of P*_lac_*, between the XbaI and PstI restriction sites of pSRKGm, the forward primer contained an in-frame stop codon (in frame with the ATG of the *lacZ* in the plasmid). Downstream of this stop codon, the primer contained the MS2 tag sequence and the first 20 nt of the as-smeR (according to the CoIP RNA-seq data). The reverse primer contained the Shine-Dalgarno sequence and the first codons of *smeR*. Since it is not clear whether as-*smeR* RNA works in *trans*, we additionally cloned a constitutive P*_sinI_* promoter driving *smeR* expression. Thus, while *smeR* is constitutively transcribed from the resulting plasmid pSRKGm-MS2as-smeR (overexpressed in the 2011 strain used), the transcription of a bicistronic *lacZ*′-MS2-as-*smeR* RNA is IPTG inducible. The *lacZ′* part of the bicistronic transcript is translated, producing a short 30-aa LacZ fragment.

Plasmids were transferred from E. coli to S. meliloti, A. tumefaciens, or B. japonicum by diparental conjugation with E. coli S17-1 as the donor (66). Bacteria were mixed, washed in saline, and spotted onto a sterile membrane filter, which was placed onto a TY plate without antibiotics. After incubation for at least 4 h (for S. meliloti and A. tumefaciens) or 3 days (for B. japonicum) at 30°C, serial dilutions were spread on agar plates with selective antibiotics.

### Nile red efflux assay.

The efflux assay was performed essentially as described (67). Cultures of strains 2011 (pSRKGm-peTrpL, pRK4352) and the EVC 2011 (pSRKGm, pRK4352) were cultivated in medium with Gm and Tc. Cultures with and without IPTG, which induces peTrpL production, were grown in parallel. Pellets from 20 ml of culture were washed in 20 mM potassium phosphate buffer (pH 7.0) containing 1 mM MgCl_2_ (PPB-Mg), and resuspended in PPB-Mg, adjusting the OD_600_ to 1.0. The cell suspension was incubated for 15 min at room temperature. Two-milliliter aliquots were transferred into glass tubes, and the efflux pump inhibitor carbonyl cyanide 3-chlorophenylhydrazone (CCCP) was added at a final concentration of 25 mM (5 mM stock solution in 50% dimethyl sulfoxide [DMSO]). After 15 min, 5 mM Nile red dye was added (a stock solution of 5 mM in 10% dimethyl formamide, 90% ethanol) and the cell suspension was incubated on a shaker (140 rpm at 30°C) for 3 h, followed by a 60-min incubation without shaking at room temperature and centrifugation for 5 min at 4,400 rpm in the tabletop centrifuge. The supernatant was entirely removed, and cells were resuspended in 1 ml PPB-Mg (or in PPB-Mg supplemented with increased Tc concentrations; Fig. S3). Immediately thereafter, 0.3 ml of this cell suspension was transferred to a 96-well microtiter plate, and 15 μl of 1 M glucose was added to trigger Nile red efflux. Fluorescence of the cell suspension was followed over 1,500 s (excitation at 552 nm and emission at 636 nm) on the Tecan reader. Three independent experiments revealed similar results.

### Analysis of the antisense promoter P_as_.

Plasmid pSUP-PasRegfp containing the transcriptional fusion of *egfp* to promoter P_as_ was used to analyze the inducibility of the promoter by antimicrobial compounds, which were added to cultures at the OD_600_ of 0.5. Since this plasmid confers Tc resistance, it was necessary to incubate strain 2011 (pSUP-PasRegfp) with the chromosomally integrated plasmid overnight without Tc (essentially all cells retained the plasmid, as confirmed by qPCR analysis) before Tc was added at the designated concentrations. Similarly, other antimicrobial compounds were added at subinhibitory concentrations (see above) to 2011 (pSUP-PasRegfp) cultures that were incubated without Tc overnight. RNA was isolated before (time point 0) and at the designated time points after antibiotic addition, and changes in the reporter *egfp* mRNA upon exposure were analyzed by qRT-PCR.

### RNA purification.

For RNA-seq or analysis of changes in RNA levels by qRT-PCR, total RNA of S. meliloti and A. tumefaciens was purified from 15 ml of culture (OD_600_ of 0.5). The cells were cooled by adding the culture directly into tubes with ice rocks (corresponding to a volume of 15 ml). After centrifugation at 6,000 × *g* for 10 min at 4°C, the pellet was resuspended in 250 μl TRIzol (Life Technologies, Darmstadt, Germany). Lysis was performed with in a laboratory mixer mill (Retsch MM200) (4°C) with glass beads, two times for 15 min, interrupted by incubation at 65°C for 10 min. Then, 750 μl TRIzol was added to the samples, and RNA was isolated according to the manufacturer’s instructions. Residual RNases were removed by additional extraction with hot phenol, phenol/chloroform/isoamyl alcohol (25:24:1) and chloroform/isoamyl alcohol (24:1). RNA was ethanol precipitated and dissolved in ultrapure water. For RNA half-life measurements by qRT-PCR and Northern blot hybridization, 1 ml S. meliloti or A. tumefaciens culture was added to 2 ml RNAprotect Bacteria reagent (Qiagen), and RNA was isolated using RNeasy columns (Qiagen). RNA from B. japonicum was isolated with hot phenol (68). For purification of RNA coimmunoprecipitated from S. meliloti, TRIzol, without subsequent hot-phenol treatment, was used. RNA from reconstituted ARNP complexes was purified using phenol/chloroform/isoamyl alcohol (25:24:1). For qRT-PCR analysis, residual DNA was removed by incubating 10 μg RNA with 1 μl TURBO-DNase (Ambion) for 30 min. Prior to the qRT-PCR analysis, the RNA samples were tested for presence of DNA by PCR with *rpoB*-specific primers.

### Northern Blot hybridization.

For analysis of ARNP RNA, samples were separated in 10% polyacrylamide-urea gels and transferred by semidry electroblotting to a positively charged nylon membrane. For total RNA analysis, 10-μg samples were separated in a 1% agarose-formaldehyde gel and vacuum blotted. Radioactive, 5′-labeled oligonucleotide probes were used to detect *in vitro* transcripts from reconstituted ARNPs (see Data Set S4). For this, the UV cross-linked membrane was prehybridized for 2 h at 56°C with a buffer containing 6× SSC (1× SSC is 0.15 M NaCl plus 0.015 M sodium citrate), 2.5× Denhardt’s solution, 1% SDS, and 10 μg/ml salmon sperm DNA. Hybridization was performed in a solution containing 6× SSC, 1% SDS, and 10 μg/ml salmon sperm DNA for at least 6 h at 56°C. Membrane washing was performed twice for 2 to 5 min in 0.01% SDS, 5× SSC at room temperature. Agarose gel blots were hybridized either with DNA probes obtained by random-primed labeling (*smeA*- and *smeB*-specific probes) or with internally labeled as-*smeR2 in vitro* transcript (see Fig. 5A). The prehybridization solution (25 ml final volume) contained 4× Denhardt’s solution, 250 mg glycine, 5× SSPE (1× SSPE is 0.18 M NaCl, 10 mM NaH_2_PO_4_, and 1 mM EDTA [pH 7.7]), 12.5 ml formamide, 0.1% SDS, and 10 μg/ml salmon sperm DNA. Hybridization was performed in a 27-ml solution containing 1× Denhardt’s solution, 5× SSPE, 13.9 ml formamide, 0.1% SDS, 2.7 g dextran sulfate, and 5 μg/ml salmon sperm DNA. Prehybridization and hybridization conditions were as described above. The washing was performed in 0.05 SDS, 1 × SSC. Signals were detected using a Bio-Rad molecular imager. For rehybridization, membranes were washed in 0.1% SDS for 20 min at 96°C.

### Radioactive labeling of hybridization probes.

Oligonucleotides (10 pmol) were labeled at the 5′ terminus using 15 μCi [γ-^32^P]ATP (Hartmann Analytics, Braunschweig, Germany) and 5 U T4 polynucleotide kinase in a 10-μl reaction mixture, which was incubated for 60 min at 37°C. After adding 30 μl water, unincorporated nucleotides were removed using MicroSpin G-25 columns (GE Healthcare Life Sciences). For preparing *smeA*- and *smeB*-specific probes, the PCR amplicon obtained with the qPCR primer (Data Set S4) was used as the template for random-primed labeling using Prime-a-Gene labeling system (Promega). The *smeR*-specific RNA probe was prepared by *in vitro* transcription (see below).

### *In vitro* transcription.

For *in vitro* transcription, the MEGAshortscript T7 kit (Thermo Fisher Scientific, Vilnius, Lithuania) was used. The T7 promoter sequence was integrated into one of the primers for PCR amplification of the template (Data Set S4), which was column-purified and eluted in ultrapure water. For nonlabeled transcripts, the reaction mixture contained 500 ng template, 1× T7 polymerase buffer, 7.5 mM ATP, 7.5 mM CTP, 7.5 mM GTP, 7.5 mM UTP, and 25 U T7 enzyme mix. For internally labeled transcripts, 0.5 mM ATP, 0.5 mM CTP, 0.5 mM GTP, and 0.1 mM UTP were used. Additionally, 2 μl [α-^32^P]UTP (10 μCi/μl) per 20-μl reaction mixture was added. After incubation for at least 5 h at 37°C, the DNA template was removed using 1 μl TURBO-DNase (1 h at 37°C). The *in vitro* transcript was extracted with acidic phenol, precipitated with ethanol, and dissolved in water.

### Strand-specific, real-time reverse transcriptase PCR.

Relative steady-state levels of specific RNAs by real-time qRT-PCR were analyzed using the Brilliant III Ultra Fast SYBR green QRT-PCR master mix (Agilent, Waldbronn, Germany). Strand-specific analysis was performed as follows: 5 μl master mix (supplied), 0.1 μl dithiothreitol (DTT) (100 mM; supplied), 0.5 μl RiboLock solution (supplied), 0.4 μl water, 1 μl of the reverse primer (10 pmol/μl), and 2 μl RNA (20 ng/μl) were assembled in a 9-μl reaction mixture. After cDNA synthesis, the reverse transcriptase was inactivated by incubation for 10 min at 96°C. Then, the samples were cooled to 4°C, 1 μl of the second primer (10 pmol) was added, and real-time PCR was performed starting with 5 min incubation at 96°C. The efficiencies of the used primer pairs (Data Set S4) were determined by PCR of serial 2-fold RNA dilutions. Primer pairs were designed using Primer3 (69). The qRT-PCRs were conducted in a spectrofluorometric thermal cycler (Bio-Rad, Munich, Germany). The quantification cycle (*C_q_*) was set to a cycle at which the curvature of the amplification is maximal (70). For determination of steady-state mRNA levels, *rpoB* (encodes the β subunit of RNA polymerase) was used as a reference gene (36). For half-life determination, the stable but highly abundant 16S rRNA was used as a reference molecule. Therefore (to achieve similar *C_q_*s of mRNA and 16S rRNA), the 10-μl reaction mixture for qRT-PCR with 16S rRNA-specific primers contained 2 μl RNA with a concentration of 0.002 ng/μl (36). The Pfaffl formula was used to calculate fold changes of mRNA amounts (71). The qRT-PCRs with an RNA sample were performed in technical replicates. If the *C_q_* difference between the technical replicates was >0.5, the analysis was repeated. In such a case, the RNA sample of the outliers and, as a control, at least one of the other RNA samples were analyzed once again by qRT-PCR. If the *C_q_* difference of the reference gene in independent biological experiments was >1 (for *rpoB*) or >2 (for 16S rRNA), the analysis was repeated. qPCR product specificity was validated by a melting curve after the qPCR and by gel electrophoresis. No-template controls and negative mRNA controls (RNAs expected to be not affected under the applied conditions, e.g., *trpDC* mRNA which is transcribed from a second *trp* operon and is regulated by rnTrpL but not by peTrpL [36]) were always included.

For analysis of total RNA, qRT-PCR of the gene of interest (e.g., *smeR*) and of the reference gene *rpoB* were performed using portions of the same DNA-free RNA sample, and log_2_ fold changes of mRNA levels after induction by IPTG and/or exposure to antibiotics were determined. Unless stated otherwise, the mRNA level after induction or exposure was compared to the level before induction or exposure. For analysis of coimmunoprecipitated RNA, the qRT-PCR of the gene of interest was performed using a CoIP RNA sample, while total RNA of the same culture (isolated from the lysate prior to adding the beads) was used for the *rpoB* qRT-PCR. Then, the Pfaffl formula was used to calculate the fold enrichment of specific RNAs by CoIP with 3×FLAG-peTrpL or 3×FLAG peptide, in comparison to the mock CoIP, which was conducted with a strain producing the nontagged peTrpL.

### mRNA half-life determination.

Stability of mRNA was determined as described (36). Ten minutes after addition of IPTG and/or Tc to cultures of strain 2011 Δ*trpL* (pSRKGm-peTrpL), rifampin was added to a final concentration of 800 μg/ml (stock concentration 150 mg/ml in methanol) to stop cellular transcription. Culture aliquots were withdrawn at time points 0, 2, 4, 6, and 8 min, and RNA was isolated using RNeasy columns. To determine the relative levels of specific mRNAs, qRT-PCR analysis with 16S rRNA as a reference was performed (see above). Additionally, Northern blot hybridization was conducted, and mRNA signals were quantified and normalized to internal control signals (16S rRNA). Linear-log graphs were used for half-life calculation.

### RNA-seq analysis.

RNA was sequenced by Vertis Biotechnologie AG (Freising, Germany). cDNA reads were mapped as described (72). Only CoIP RNA from beads washed with Tc-containing buffer was subjected to RNA-seq analysis.

### Real-time PCR.

Plasmid-specific primers (Data Set S4) were used to test whether the chromosomally integrated plasmid pSUP-PasRegfp is lost after culture incubation without selective pressure overnight. As a reference gene, *rpoB* was used. Power SYBR PCR master mix (Qiagen) was used for the qPCRs. The template and primer concentrations, reaction conditions, and quantification were performed as described for qRT-PCR of total RNA.

### Coimmunoprecipitation using 3×FLAG-peTrpL.

The CoIP of RNA that was used for RNA-seq analysis was performed with the two-plasmid strain 2011 (pSRKGm-3×FLAG-peTrpL, pRK4352) which was cultivated in medium with Gm (10 μg/ml) and Tc (20 μg/ml). Cells were harvested 10 min after induction of 3×FLAG-peTrpL production with IPTG. For a control addressing whether the 3×FLAG peptide interacts with the RNAs of interest, strain 2011 (pSRKGm-3×FLAG, pRK4352) was used. In parallel, strain 2011 (pSRKGm-peTrpL, pRK4352) was cultivated and treated similarly (mock CoIP control). Cell pellets were resuspended in 5 ml buffer A (20 mM Tris [pH 7.5], 150 mM KCl, 1 mM MgCl_2_, 1 mM DTT) containing 10 mg/ml lysozyme, 2 μg/ml Tc, and 1 tablet of protease inhibitor cocktail (Sigma-Aldrich, St. Louis, MO, USA) per 40 ml buffer. After lysis by sonication, 40 μl anti-FLAG M2 magnetic beads (Sigma-Aldrich, catalog number SLBT7133) was added to the cleared lysate and incubated for 2 h at 4°C. Then, the beads were split into two portions: one of them was washed 3 times with 500 μl buffer A containing 2 μg/ml Tc, while the other was washed with buffer without Tc. Protease inhibitors were included in the first two washing steps. Finally, the beads were resuspended in 50 μl buffer A and used for RNA purification, SDS-PAGE analysis, or mass spectrometry.

One-plasmid strains containing pSRKGm derivatives were also used for CoIP after exposure to subinhibitory concentrations of Tc or designated antimicrobial compounds. Strain 2011 (pSRKGm-3×FLAG-peTrpL) and the corresponding mock control 2011 (pSRKGm-peTrpL) were cultivated in medium with Gm only. FLAG-CoIP was conducted 10 min after addition of an antibiotic or flavonoid to the cultures along with IPTG. The same subinhibitory concentrations of antimicrobial compounds were used in the washing buffer of the CoIP procedure.

### ARNP complex reconstitution.

The peTrpL and 3×FLAG-peTrpL peptides that were used were synthesized by Thermo Fisher Scientific (Darmstadt, Germany). Ten milligrams peTrpL was dissolved in 50 μl acetonitrile, and 950 μl ultrapure water was added. One milligram 3×FLAG-peTrpL was dissolved in 1 ml 50% DMSO; 50-μl aliquots were stored at −20°C. Peptides were diluted in ultrapure water prior to usage. For reconstitution, 100 ng mini-*smeR in vitro* transcript (4.4 pmol), 100 ng antisense *in vitro* transcript (4.4 pmol), 50 ng peTrpL (27 pmol), and 50 ng 3×FLAG-peTrpL (11 pmol) were mixed in buffer B (20 mM Tris [pH 8.0], 150 mM KCl, 1 mM MgCl_2_, 1 mM DTT), in a volume of 48 μl. Then, 2 μl antibiotic or flavonoid solution was added. To negative control samples, 2 μl ethanol, methanol, or water (the solvents of the antibiotic solutions) was added. The following final concentrations of the antimicrobial compounds were used: 1.5 μg/ml Tc, 27 μg/ml Em, 9 μg/ml Cl, 45 μg/ml Km, 90 μg/ml Gs, and 45 μg/ml Lt. The samples were incubated for 20 min at 20°C under shaking, and then 3×FLAG-peTrpL-containing complexes were isolated by CoIP with anti-FLAG antibodies. The antimicrobial compounds were present in the washing buffer in the concentrations given above. After extensive washing, RNA was purified and analyzed by Northern blotting hybridization.

### EMSAs.

For gel-shift assays, 100 ng of internally radiolabeled *in vitro* transcript was denatured at 95°C and mixed with WT or mutated synthetic peTrpL peptides (0 to 100 ng), 1 μl RiboLock, and 1.5 μg/ml Tc in the reconstitution buffer B, in a final volume of 20 μl. When appropriate, a complementary transcript was added after the denaturing step. The samples were incubated for 20 min at 20°C under shaking. After adding 2 μl of loading buffer (0.05× Tris-borate-EDTA [TBE], 50% glycerol, 0.1% bromphenol blue, 1.5 μg/ml Tc), the samples were loaded onto a 2-mm-thick, 10% native polyacrylamide gel (10% PAA, 0.25× TBE, 10 mM MgCl_2_, 1.5 μg/ml Tc). The electrophoretic separation was conducted for 3 h at 150 V and 4°C. Gel was prerun for 1 h at 100 V and 4°C. For EMSA in the absence of Tc, instead of Tc, the same volume of the solvent ethanol was added to the reconstitution samples and to the gel. After gel drying, signals were detected by phosphorimaging.

### Isolation of MS2-as-smeR RNA by MS2-MBP affinity chromatography.

For MS2-MBP affinity chromatography, amylose beads were noncovalently bound to the MS2 coat protein fused to maltose-binding protein (MS2-MBP), which was purified from E. coli, as described (43). Ten minutes after IPTG addition to S. meliloti 2011 (pSRKGm-MS2-as-smeR, pSRKTc-3×FLAG-peTrpL) cultures, cells were harvested. Chromatography was performed as described (43) with the following modification. For washing, the beads were split into two portions, and one of them was washed with buffer B (20 mM Tris [pH 8.0], 150 mM KCl, 1 mM MgCl_2_, 1 mM DTT) containing 2 μg/ml Tc, while the second one was washed with the buffer without Tc. The 3×FLAG-peTrpL and peTrpL peptides of the elution fractions were analyzed by mass spectrometry.

### SDS-PAGE and Western blot analysis.

Glycine- and tricine-SDS-PAGE were conducted as described (61, 73). For Tricine-SDS gels, 16% polyacrylamide separating gel (acrylamide/bisacrylamide [19:1]; Carl Roth, Karlsruhe, Germany) containing 8% glycerol was used. Detection of FLAG-tagged proteins transferred onto a polyvinylidene difluoride (PVDF) membrane (GE Healthcare Life Sciences) was performed with monoclonal anti-FLAG M2-horseradish peroxidase (HRP) antibodies (Sigma-Aldrich) and a Lumi-Light Western blotting substrate kit (Roche, Basel, Switzerland).

### Mass spectrometry.

For identification of proteins in a gel slice stained with Coomassie brilliant blue, the band was destained and digested with trypsin as reported elsewhere (74). To recover the peptides, gel pieces were covered with ultrapure water and incubated 15 min in an ultrasonic water bath. Peptides derived from in-gel digestion were loaded on an EASY-nLC II system (Thermo Fisher Scientific) equipped with an in-house built 20-cm column (inner diameter, 100 mm; outer diameter, 360 mm) filled with ReproSil-Pur 120 C_18_-AQ reversed-phase material (3-mm particles, Dr. Maisch GmbH). Elution of peptides was executed with a nonlinear 80-min gradient from 1% to 99% (vol/vol) solvent B (0.1% [vol/vol] acetic acid in acetonitrile) with a flow rate of 300 nl/min and injected online into an LTQ Orbitrap XL (Thermo Fisher Scientific). The survey scan at a resolution of *R* = 30.000 and 1 × 10^6^ automatic gain control target in the Orbitrap with activated lock mass correction was followed by selection of the five most abundant precursor ions for fragmentation. Singly charged ions as well as ions without detected charge states were excluded from tandem mass spectrometry (MS/MS) analysis.

For quantification of peTrpL abundance by targeted MS, protein extracts were diluted in 50 mM triethylammonium bicarbonate (TEAB) buffer (pH 8.0; Sigma-Aldrich) to a final concentration of 0.5 μg/μl. After protein reduction (2.5 mM tris-(2-carboxyethyl)phosphine hydrochloride [TCEP]; Invitrogen) at 65°C for 45 min, thiols were alkylated in 5 mM iodoacetamide (Sigma-Aldrich) for 15 min at 25°C in the dark. For protein digestion, trypsin (Promega) was added in an enzyme-to-substrate ratio of 1:100. After 14 h at 37°C, digestion was terminated by adding concentrated HCl to a final concentration of 600 mM, and peptides were purified by C_18_ Zip tips (Pierce). Prior measurement samples were spiked with synthetic peptides containing an isotopically labeled amino acid (JPT Peptide Technologies and Thermo Fisher Scientific) to a final concentration of 50 fmol/μl (FLAG-peTrpL), 100 fmol/μl (peTrpL-M), and 1,000 fmol/μl (WT peTrpL). For quantification of peTrpL abundance, the heavy synthetic peptide was used to optimize MS parameters to achieve the highest sensitivity. The samples were loaded on an EASY-nLC 1000 or an EASY-nLC II system (Thermo Fisher Scientific) equipped with an in-house built 20-cm column (see above). Elution of peptides was executed with a nonlinear gradient from 1% to 99% (vol/vol) solvent B (0.1% [vol/vol] acetic acid in acetonitrile) with a flow rate of 300 nl/min and injected online into a TSQ Vantage (Thermo Fisher Scientific). The selectivity for both Q1 and Q3 were set to 0.7 Da (full width at half maximum [FWHM]). The instrument was operated in SRM mode applying a collision gas pressure of 1.2 mTorr in Q2. All monitored transitions and the optimized collision energy can be found in Data Set S5.

10.1128/mBio.01027-20.10 DATA SET S5 Precursor ion masses (Q1 *m/z*), fragment ion masses (Q3 *m/z*), the optimized collision energy and all monitored transitions are provided for the quantified peTrpL peptide in both isotope forms. Download Data Set S5, XLSX file, 0.1 MB. Copyright © 2020 Melior et al. 2020 Melior et al. This content is distributed under the terms of the Creative Commons Attribution 4.0 International license.

### Processing of mass spectrometry data.

For identification of peptides from MS spectra, a database search was performed with Sorcerer-Sequest (4.0.4 build, Sage-N Research) using the Sequest algorithm against a target decoy-integrated proteogenomic database (iPtgxDB; https://iptgxdb.expasy.org/), which also contained sequences of common laboratory contaminants and FLAG-tagged peTrpL (total entries, 320,482). The S. meliloti 2011 iPtgxDB was created by integrating and consolidating the annotations of the chromosome (NC_020528) and two plasmids (NC_020527 and NC_020560) from RefSeq (75) and Genoscope (76), with predictions from Prodigal (77), ChemGenome (78) and a modified form of six-frame predicted ORFs (79). The database search was based on a strict trypsin digestion with two missed cleavages permitted. Oxidation of methionine and carbamidomethylation of cysteine were considered variable modifications. The mass tolerance for precursor ions was set to 10 ppm, and the mass tolerance for fragment ions was set to 0.5 Da. Validation of MS/MS-based peptide and protein identification was performed with Scaffold V4.7.5 (Proteome Software, Portland, OR, USA), and peptide identifications were accepted if they exceeded the following thresholds: deltaCn greater than 0.1 and XCorr scores greater than 2.2, 3.3, and 3.75 for doubly, triply, and all higher charged peptides, respectively. Protein identifications were accepted if at least 2 identified peptides were detected for proteins with a molecular weight of 15 kDa and higher. For proteins smaller than 15 kDa, the identification of one unique peptide fulfilling the criteria mentioned above was sufficient for an identification. Normalized spectrum abundance factors (80) were used as proxy for protein abundance in the sample.

All raw files from targeted MS were processed using Skyline 4.2 (81). A peptide ratio of native and heavy species was based on five transitions. Peptide ratios based on a Dot-Product of >0.7 were used to calculate the average from three biological replicates. The concentration of native peptides in the sample was calculated based on the peptide ratios and the added amount of heavy peptide.

### EGFP fluorescence measurement.

Fluorescence of strains producing peTrpL′-enhance green fluorescent protein (EGFP) fusion protein or EGFP was measured using a Tecan Infinite M200 reader. Fluorescence of EVC strains that do not harbor *egfp* was also measured. Values were normalized to the ODs measured on the Tecan. The EVC values were subtracted from the values of the peTrpL′-EGFP- or EGFP-producing cultures.

### Analysis of the conservation of peTrpL function.

Phyre^2^ (42) was used to analyze SmeR, leading to its identification as a TtgR (8) homolog. The closest homologs of the S. meliloti *smeR* gene in A. tumefaciens and B. japonicum were identified by BLASTP (https://blast.ncbi.nlm.nih.gov/Blast.cgi). In A. tumefaciens, this was the gene Atu3201 (*acrR*), which encodes a TetR-type repressor and is a part of the *acrABR* operon (13). In B. japonicum, the best match was the orphan gene blr2396 encoding a TetR-type repressor, and this gene was analyzed. Plasmid pSRKTc-Atu-peTrpL was used to induce by IPTG the Atu-peTrpL production in A. tumefaciens for 10 min. Changes in mRNA levels were calculated in comparison to that at 0 min. Due to the lack of a suitable inducible system for B. japonicum, Bja-peTrpL was overproduced constitutively from the chromosomally integrated Tc resistance-conferring plasmid pRJ-Bja-rnTrpL. Changes in mRNA levels were calculated in comparison to the EVC. Phenotypic changes were tested as indicated.

### Data availability.

The RNA-seq and RNA immunoprecipitation sequencing (RIP-seq) data discussed in this publication have been deposited in NCBI’s Gene Expression Omnibus (82) under accession number GSE118689. The MS data discussed in this publication have been deposited to the ProteomeXchange Consortium via the PRIDE partner repository (83) with the data set identifier PXD018342. The S. meliloti 2011 iPtgxDB is available at https://iptgxdb.expasy.org/database/.
