# Supplementary figures and images for: The Leader Peptide peTrpL Forms Antibiotic-Containing Ribonucleoprotein Complexes for Posttranscriptional Regulation of Multiresistance Genes
Source: mBio. 2020 Jun 16;11(3):e01027-20. doi: 10.1128/mBio.01027-20 (PMC7298713; doi:10.1128/mBio.01027-20)

# Figure S1

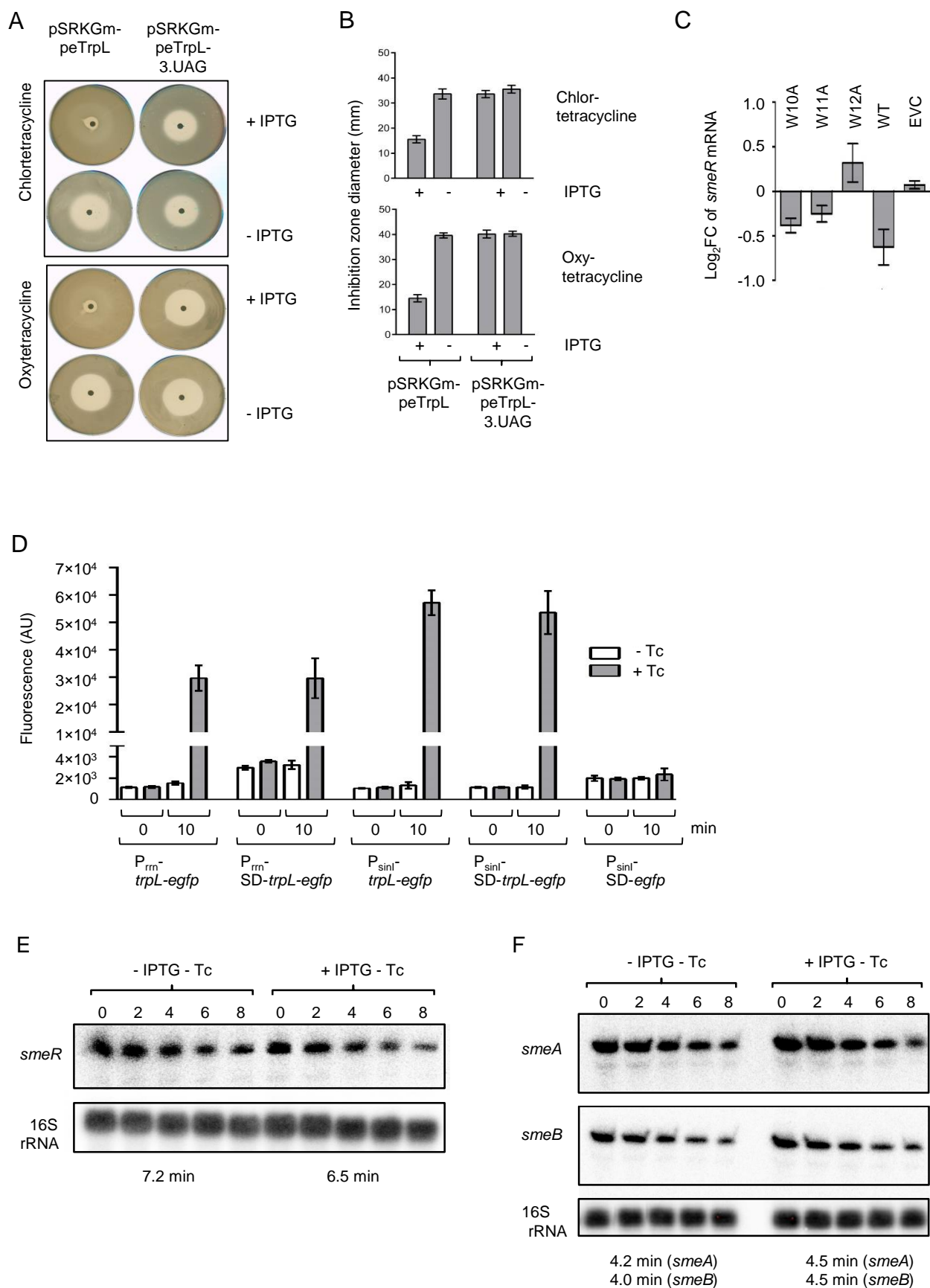

Supplement: FIG S1 [file mBio.01027-20-sf001.pdf]

Figure S2

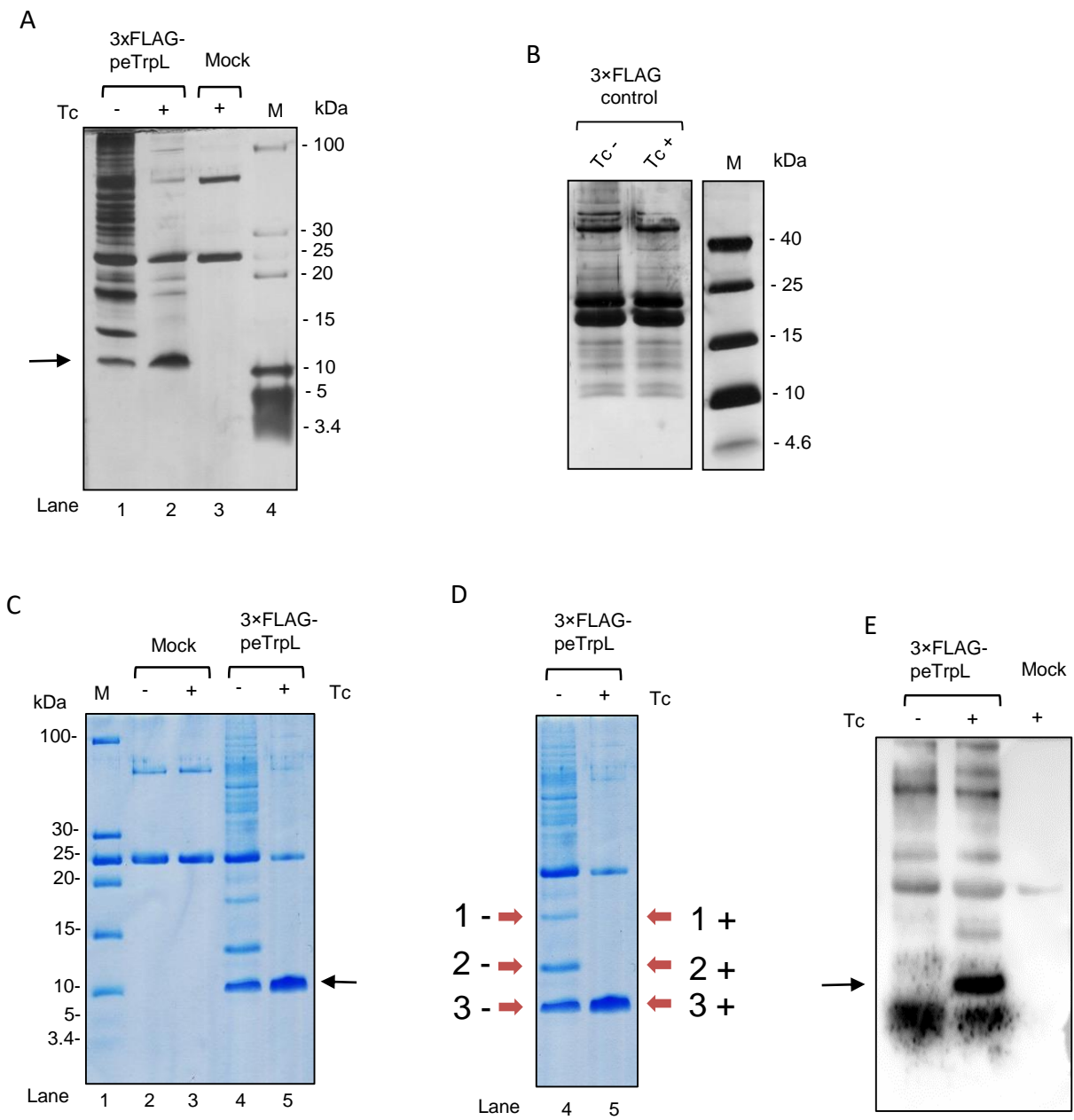

Supplement: FIG S2 [file mBio.01027-20-sf002.pdf]

Figure S3

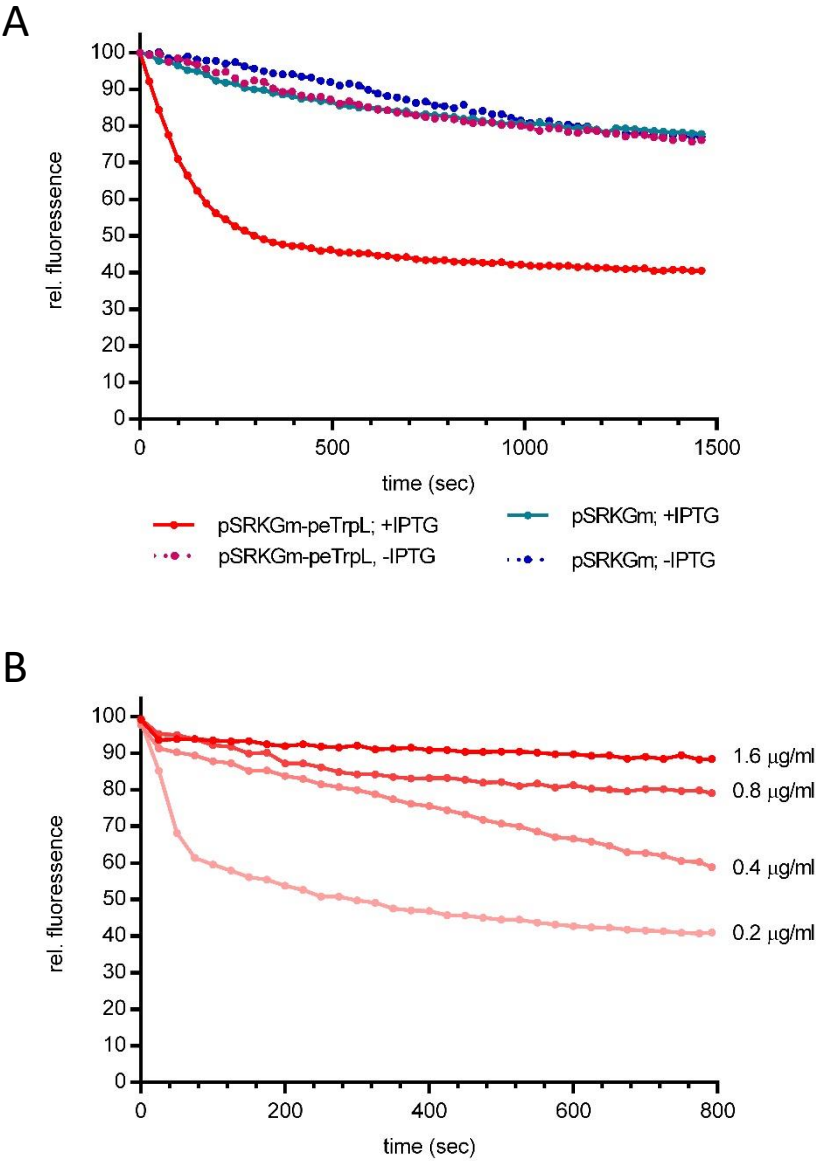

Supplement: FIG S3 [file mBio.01027-20-sf003.pdf]

Figure S4

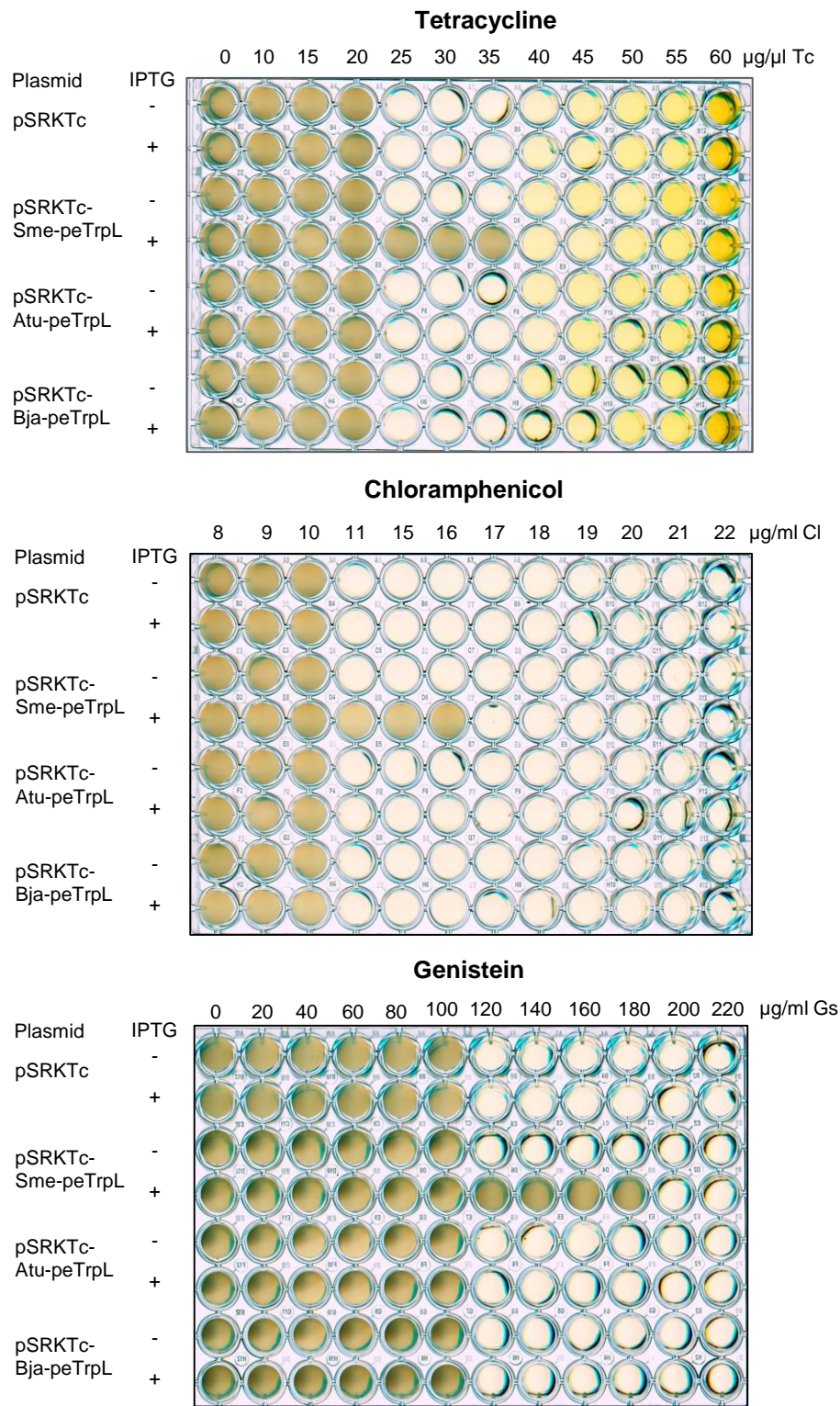

Supplement: FIG S4 [file mBio.01027-20-sf004.pdf]
