# Supplementary material for: The Leader Peptide peTrpL Forms Antibiotic-Containing Ribonucleoprotein Complexes for Posttranscriptional Regulation of Multiresistance Genes
Source: mBio. 2020 Jun 16;11(3):e01027-20. doi: 10.1128/mBio.01027-20 (PMC7298713; doi:10.1128/mBio.01027-20)
Supplement: TABLE S1 [file mBio.01027-20-st001.pdf]

Table S1. Strains and plasmids used in this work.

Strains	Ref.	Description
Sinorhizobium meliloti 2011	[1]	Strain Rm2011, contains insertion sequence within *expR* gene, parental strain in this study; Sm^r.
Sinorhizobium meliloti 2011*ΔtrpL*	[2]	*ΔtrpL* mutant of strain 2011; Sm^r.
Agrobacterium tumefaciens NTL4	[3]	Used for analysis of Atu-peTrpL.
Bradyrhizobium japonicum USDA 110spc4	[4]	Used for analysis of Bja-peTrpL; Sp^r.
Escherichia coli DH5α	Thermo Fisher	Used for cloning; *fhuA2 lac(del)U169 phoA glnV44 Φ80' lacZ(del)M15 gyrA96 recA1 relA1 endA1 thi-1 hsdR17*.
Escherichia coli S17-1	[5]	*E. coli* 294 Thi RP4-2-Tc::Mu-Km::Tn*7* integrated into the chromosome.
**Plasmids**	**Ref.**	**Description**
pRK4352	[6]	Broad host range plasmid with *rrn* promoter from *Rhodobacter sphaeroides*; Tc^r.
pRK-rnTrpL (synonym pRK-SmelRcsR1)	[7]	pRK4352 derivative for constitutive production of rnTrpL (previously SmelRcsR1) and peTrpL; Tc^r.
pRK-trpL-egfp	[2]	pRK4352 derivative; contains a translational *S. meliloti trpL::egfp* fusion harboring the first 6 codons of *trpL* fused to the third codon of *egfp*; the *trpL::egfp* transcript is leaderless; Tc^r
pRK-SDtrpL-egfp	This work	pRK4352 derivative similar to pRK-trpL-egfp, with exception of a typical Shine-Dalgarno sequence in the transcript, upstream of the *trpL* start codon; Tc^r.
pSRKTc	[8]	Broad host range expression vector with tightly regulated, IPTG inducible *lacZ* promoter; Tc^r
pSRKTc-peTrpL	This work	pSRKTc derivative; contains a recombinant ORF encoding the *S. meliloti* peTrpL peptide, cloned in frame with the ATG of the NdeI restriction site; Tc^r.
pSRKTc-Atu-peTrpL	This work	pSRKTc derivative; contains a recombinant ORF encoding the *A. tumefaciens* peTrpL peptide, cloned in frame with the ATG of the NdeI restriction site; Tc^r.
pSRKTc-Bja-peTrpL	This work	pSRKTc derivative; contains a recombinant ORF encoding the *B. japonicum* peTrpL peptide, cloned in frame with the ATG of the NdeI restriction site; Tc^r.
pSRKGm	[8]	Broad host range expression vector with tightly regulated, IPTG inducible *lacZ* promoter; Gm^r
pSRKGm-peTrpL	This work	pSRKGm derivative; contains a recombinant ORF encoding the *S. meliloti* peTrpL peptide, cloned in frame with the ATG of the NdeI restriction site; Gm^r.
pSRKGm-peTrpL-3.UAG	This work	pSRKGm-peTrpL derivative, in which the third codon is replaced by a stop codon; Gm^r.
pSRKGm-peTrpL-W10A	This work	pSRKGm-peTrpL derivative, in which the 10. (tryptophan) codon is replaced by an alanine codon; Gm^r.
pSRKGm-peTrpL-W11A	This work	pSRKGm-peTrpL derivative, in which the 11. (tryptophan) codon is replaced by an alanine codon; Gm^r.
pSRKGm-peTrpL-W12A	This work	pSRKGm-peTrpL derivative, in which the 12. (tryptophan) codon is replaced by an alanine codon; Gm^r.
pSRKGm-3×FLAG-peTrpL	This work	pSRKGm-peTrpL derivative for production of 3×FLAG-peTrpL; Gm^r.
pSRKGm-3×FLAG-peTrpL-W10A	This work	pSRKGm-3×FLAG-peTrpL derivative, in which the 10. (tryptophan) codon is replaced by an alanine codon; Gm^r.
pSRKGm-3×FLAG-peTrpL-W12A	This work	pSRKGm-3×FLAG-peTrpL derivative, in which the 12. (tryptophan) codon is replaced by an alanine codon; Gm^r.
pSRKGm-3×FLAG	This work	pSRKGm derivative, contains an ORF encoding a 3×FLAG peptide; Gm^r.
pSRKGm-MS2as-smeR	This work	pSRKGm derivative; used for IPTG-induced transcription of MS2-as-smeR RNA; Gm^r.

pSRKTc-3×FLAG-peTrpL	This work	pSRKTc-peTrpL derivative for production of 3×FLAG-peTrpL; Tc^r.
pSUP202pol4	[9]	Suicide plasmid, allows for construction of integration vectors; Tc^r.
pSUP202pol4-exoP	This work;	pSUP202pol4 derivative, contains 300 nt of the 3′ *exoP* region as an chromosomal integration site in *S. meliloti* [10]; Tc^r.
pSUP-PasRegfp	This work	pSUP202pol4-exoP derivative, contains a transcriptional fusion of *egfp* to the promoter P_{as} (-290 to +2 in respect to the putative asRNA transcription start site, as determined by RNAseq of CoIP RNA); the *egfp* ORF is preceded by a Shine-Dalgarno sequence, Tc^r.
pSW2	S.B.W and E.E.H, unpublished	pSRKGm derivative, in which the *E. coli lacZ* operon module was replaced by the constitutive promoter P_{sinI} followed by XbaI and BamHI restriction sites and a transcription terminator; Gm^r.
pSW2-trpL-egfp	This work	pSW2 derivative, allows for constitutive transcription of the leaderless *trpL::egfp* fusion from P_{sinI}; the fusion contains the first 6 codons of *trpL* fused to the third codon of *egfp*; Gm^r.
pSW2-SDtrpL-egfp	This work	Similar to pSW2-trpL-egfp, but the constitutively transcribed *trpL::egfp* is preceded by a Shine-Dalgarno sequence; Gm^r
pRS-SDegfp	This work	pSW2 derivative, in which *egfp* preceded by a Shine-Dalgarno sequence is constitutively transcribed from P_{sinI}; kindly provided by Robina Scheuer ; Gm^r.
pRJ-MCS (synonym pRJPaph-MCS)	[11]	Integration vector for *B. japonicum*; contains a constitutive *nptII* promoter; Tc^r
pRJ-Bja-peTrpL	This work	pRJ-MCS derivative for constitutive overproduction of peTrpL in *B. japonicum*; Tc^r
pLK46	[12]	Contains *egfp* with GC rich codons; Tc^r

References:

1. Casse F, Boucher C, Julliot JS, Michel M, Denarie J. 1979. Identification and characterization of large plasmids in *Rhizobium meliloti* using agarose-gel electrophoresis. Gen Microbiol 113: 229-242.
2. Melior H, Li S, Madhugiri R, Stötzel M, Azarderakhsh S, Barth-Weber S, Baumgardt K, Ziebuhr J, Evguenieva-Hackenberg E. 2019. Transcription attenuation-derived small RNA rnTrpL regulates tryptophan biosynthesis gene expression in *trans*. *Nucleic Acids Res* 47: 6396-6410.
3. Cha C, Gao P, Chen YC, Shaw PD, Farrand SK. 1998. Production of acyl-homoserine lactone quorum-sensing signals by gram-negative plantassociated bacteria. Mol Plant Microbe Interact 11: 1119-1129.
4. Regensburger B, Hennecke H. 1983. RNA polymerase from *Rhizobium japonicum*. Arch Microbiol 135: 103-109.
5. Simon R, Priefer U, Pühler A. 1982. A broad host range mobilization system for *in vivo* genetic engineering: transposon mutagenesis in gram-negative bacteria. Biotechnology 1: 784-791.

6. Mank NN, Berghoff BA, Hermanns YN, Klug G. 2012. Regulation of bacterial photosynthesis genes by the small noncoding RNA PcrZ. Proc. Natl Acad Sci U S A 109**:** 16306-16311.
7. Baumgardt K, Šmídová K, Rahn H, Lochnit G, Robledo M, Evguenieva-Hackenberg E (2016) The stress-related, rhizobial small RNA RcsR1 destabilizes the autoinducer synthase encoding mRNA *sinI* in *Sinorhizobium meliloti*. *RNA Biol* 13**:** 486-499.
8. Khan SR, Gaines J, Roop RM2[nd], Farrand SK. 2008. Broad-host-range expression vectors with tightly regulated promoters and their use to examine the influence of TraR and TraM expression on Ti plasmid quorum sensing. Appl Environ Microbiol 74: 5053-5062.
9. Fischer HM, Babst M, Kaspar T, Acuna G, Arigoni F, Hennecke H. 1993. One member of a gro-ESL-like chaperonin multigene family in *Bradyrhizobium japonicum* is co-regulated with symbiotic nitrogen fixation genes*.* EMBO J 12: 2901-2912.
10. Schlüter JP, Czuppon P, Schauer O, Pfaffelhuber P, McIntosh M, Becker A. 2015. Classification of phenotypic subpopulations in isogenic bacterial cultures by triple promoter probing at single cell level. J Biotechnol 198: 3-14.
11. Hahn J, Thalmann S, Migur A, von Boeselager RF, Kubatova N, Kubareva E, Schwalbe H, Evguenieva-Hackenberg E. 2017. Conserved small mRNA with an unique, extended Shine-Dalgarno sequence. RNA Biol 14: 1353-1363.
12. McIntosh M, Krol E, Becker A. 2008. Competitive and cooperative effects in quorum-sensing-regulated galactoglucan biosynthesis in *Sinorhizobium meliloti*. J Bacteriol 190: 5308-5317.
